# Ordered Chaos: Photoreceptor Mosaics in the Larval and Adult Turquoise Killifish (*Nothobranchius furzeri*)

**DOI:** 10.1002/cne.70199

**Published:** 2026-09-13

**Authors:** Nicole C. L. Noel

**Affiliations:** ^1^ Institute of Ophthalmology University College London London UK; ^2^ School of Optometry and Vision Science University of Waterloo Waterloo Ontario Canada

**Keywords:** AB_10013805, AB_3212350, cone photoreceptors, opsins, retinal regionalization, rod photoreceptors, teleost, vision

## Abstract

An organism's vision is dependent on the photoreceptor types in the eye and how these cells are organized across the retina. The turquoise killifish (*Nothobranchius furzeri*) is an annual killifish species that lives in the turbid waters of ephemeral pools. Although turquoise killifish are gaining popularity as a model, we know little about their photoreceptors. Here, the turquoise killifish retina was investigated for photoreceptor subtype identity and mosaic organization at the adult and larval stages to assess specialization for their light environment. Turquoise killifish were determined to have cone‐dominant retinas, with expression of UV, blue, green, and red cone opsins, as well as rhodopsin. Adult fish were found to have an ordered square mosaic, wherein double cones formed squares with single cones in the center and corners of the squares. Double cones could be comprised of a red and green or two red cone units, while the single cones could be UV, blue, red, or — very rarely — green in identity. Although ordered, the mosaic did not have regularity of cone subtype orientation across the retina. Larvae were found to have retinal regionalization, with a dorsonasal region of increased short‐wavelength cone density. In both larvae and adult retinas, red cones were the predominant cone subtype. This study presents the first description of the photoreceptor types and mosaic in an annual killifish, and provides a foundation for future work investigating killifish photoreceptor biology and visual function.

## Graphical abstract

The annual turquoise killifish (*Nothobranchius furzeri*) cone photoreceptor mosaic is an ordered lattice but lacks regularity of cone subtypes in adulthood. The cone mosaic differs between life stages and has characteristics observed in other fishes adapted for life in turbid water.

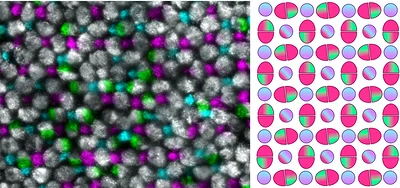

## 1 Introduction

The types of vision an organism has are determined by the complement of light‐detecting photoreceptor cells found within its retina and how those cells are organized across the tissue. This organization is referred to as a photoreceptor mosaic, and broadly, there are three categories of mosaics: stochastic mosaics, in which different cell types appear without any observable regionalization or pattern; regionalized mosaics, whereby densities of specific photoreceptor types predictably differ across the retina (though organization within these regions may be stochastic); and ordered mosaics, which have cells organized in predictable patterns, often rows or squares, although pentagonal and hexagonal patterns have also been observed (Engström 1960, 1961, 1963a; Reckel et al. 2002; Collin and Shand 2008; Viets et al. 2016; Carleton et al. 2020). Retinas can combine features of these archetypal mosaics, and mosaic organization can differ between an organism's life stages to reflect different visual niches (Archer et al. 1995; Fadool 2003; Allison et al. 2010; Yoshimatsu et al. 2020; Bolstad and Novales Flamarique 2023).

Photoreceptor mosaic organization impacts how an organism sees the world and thereby influences what behaviors they exhibit in response to visual stimuli. An example is the human retina, which has a regionalized mosaic where distinct regions have different photoreceptor types and distribution; the peripheral retina is extremely rod‐rich with red, green, and blue cones studded throughout, while the central retina—the macula—is comparatively cone‐rich, with the central foveal pit containing exclusively red and green cone photoreceptors (Samy and Hirsch 1989; Hofer et al. 2005). This concentration of cones in the central retina facilitates high‐acuity central vision, allowing for humans to perform tasks such as reading. Conversely, zebrafish (*Danio rerio*) have an ordered mosaic—specifically, the adult zebrafish photoreceptors are organized in rows, with alternation of cone subtypes within the rows (Engström 1960; Tohya et al. 1999, 2003; Raymond and Barthel 2004; Allison et al. 2010; Noel and Allison 2018). Although the photoreceptors are organized into a predictable lattice, the adult zebrafish retina also has regionalized properties in terms of photoreceptor opsin paralog expression and density (Takechi and Kawamura 2005). Larval zebrafish mosaics have different proportions of rods and cone subtypes, lack the structured row organization of the adult retina, and have notable regionalization with differences in photoreceptor density (Fadool 2003; Allison et al. 2010; Yoshimatsu et al. 2020). A UV cone‐dense region allows for high‐acuity vision in the larval zebrafish, responsible for visually mediated behaviors such as prey capture (Yoshimatsu et al. 2020).

A diversity of fish species have been investigated for broad photoreceptor mosaic pattern (Eigenmann and Shafer 1900; McEwan 1938; Engström 1960, 1961, 1963a, 1963b; Engström and Ahlbert 1963; Downing et al. 1986; Rossetto et al. 1992; Easter and Cameron 1993; Beaudet et al. 1997; Nishiwaki et al. 1997; Reckel et al. 2002; Pointer et al. 2005; Cheng and Flamarique 2007; Mokhtar et al. 2024), although the detailed organization of cone subtypes within the mosaic has only been assessed in a subset of species. Diurnal teleost fishes often have four cone subtype classes—ultraviolet (UV; *sws1* genes), blue (*sws2* genes), green (*rh2* genes), and red (*lws* genes) light‐sensitive—with an average of six cone opsin genes due to gene duplications (Vihtelic et al. 1999a; Helvik et al. 2001b; Carleton et al. 2020). Photoreceptor opsin paralogs expressed, mosaic composition, and mosaic organization can differ substantially between life stages in fishes. Larval fish typically have extremely cone‐dominant retinas, and young larval retinas often have few or no rod photoreceptors, and the rod photoreceptors present are nonfunctional until later stages (Sandy and Blaxter 1980; Pankhurst 1983; Nawrocki et al. 1985; Helvik et al. 2001a; Fadool 2003; Fleisch et al. 2008). While larval retinas are comprised solely of single cones, in adults, there are both single and physically fused cones—typically double cones consisting of two members, but rarely triple or quadruple cones (Lyall 1957)—which form ordered lattices. Short‐wavelength‐sensitive UV or blue opsins are expressed in single cones, whereas medium‐ and long‐wavelength‐sensitive green and red opsins are expressed in physically fused cones as well as single cones of some species. Nocturnal and deep‐water fishes with rod‐dominant retinas often have cone mosaics that are broken down and lack the order of diurnal cone mosaics, sometimes referred to as “disintegrated” (Engström 1963a; Carleton et al. 2020; Frau et al. 2020; De Busserolles et al. 2021).

Annual killifishes are adapted to live in temporary water bodies, with drought‐resistant embryos that can enter a developmental stasis called diapause and rapid sexual maturity post hatching. Photoreceptor complements and organization have not been investigated in annual killifish species, and thus what adaptations they may have to their environments is not known, nor how their retinas change between life stages. Turquoise killifish (*Nothobranchius furzeri*) are an annual killifish that live in turbid ephemeral pools. A previous transcriptomics study of turquoise killifish identified rod and cone populations within the killifish retina (Bergmans et al. 2024)—however, the cone photoreceptor subtypes, proportions, and mosaic organization have yet to be investigated. Here, I aimed to investigate the turbid water‐dwelling annual turquoise killifish for retinal regionalization and potential cone mosaic adaptations for living in water with diminished clarity. I characterized the photoreceptor proportions and cone mosaic in free‐feeding larval (1 day post hatching) and post‐metamorphosis sexually mature young adult (6 weeks post hatching) turquoise killifish to assess changes between major life stages. It was found that both larval and adult turquoise killifish retinas are cone‐dominant and enriched for red light‐sensitive cone photoreceptors. This work is foundational for future studies aiming to investigate visual function in the turquoise killifish or other annual killifishes, and contributes to our understanding of how photoreceptor mosaic specialization differs across species living in different light conditions.

## 2 Methods

### 2.1 Animal Care and Maintenance

Gonarezhou (GRZ) strain turquoise killifish were used for these studies. Fish were maintained at University College London on a 14:10 light/dark cycle. All experimental procedures were carried out in accordance with the UK Home Office Animals (Scientific Procedures) Act 1986 under PPLs 70/7391 and PP2133797. Embryos were kept at 28°C in Ringers solution until pigmentation developed, then transferred onto damp coconut fiber until hatching at the golden eye stage, when golden colored iridophores uniformly cover the eye. Hatching was induced by humic acid treatment as described in the literature (Chen et al. 2023).

### 2.2 Phylogenetics

Coding sequences for *sws1*, *sws2*, *rh2*, *lws*, *rho*, and *opn4a* genes in diverse teleost fishes—turquoise killifish (*N. furzeri*), zebrafish (*D. rerio*), goldfish (*Carassius auratus*), common carp (*Cyprinus carpio*), Atlantic salmon (*Salmo salar*), rainbow trout (*Oncorhynchus mykiss*), Nile tilapia (*Oreochromis niloticus*), shortfin molly (*Poecilia mexicana*), and sheepshead minnow (*Cyprinodon variegatus*)—were obtained using NCBI and Ensembl. The *opn4a* gene was used as an outgroup to root the tree, as a nonvisual opsin. Nucleotide MUSCLE alignments (https://www.ebi.ac.uk/jdispatcher/msa/muscle) were performed for the sequences. Alignments were uploaded into the maximum likelihood tree‐generating software IQ‐Tree (https://iqtree.github.io, v1.6.12) (Nguyen et al. 2015), using the best‐fit model TPM2+F+I+G4 with 1000 bootstrap inferences. Finalized trees were visualized and formatted using Interactive Tree of Life (iTOL; https://itol.embl.de).

### 2.3 Tissue Preparation

Young adult (6 weeks post hatching) or 1 day post hatching larval killifish were used for these experiments. For cryosectioning, eyes were enucleated and fixed in 4% paraformaldehyde + 5% sucrose overnight at 4°C, then transferred into 30% sucrose in PBS overnight at 4°C to cryoprotect the tissue. Eyes were then embedded in 3% agarose, excess agarose trimmed away, and returned to 30% sucrose until the eyes sank. Tissue was then embedded in polyfreeze tissue freezing medium (Sigma Aldrich, Cat. No. SHH0025) and stored at −70°C before sectioning on a Leica CM1950 cryostat at a thickness of 10–12 µm. Tissue was allowed to dry on slides for 20 min at room temperature, and slides were stored at −70°C. Before use, slides were allowed to warm to room temperature for 20 min followed by tissue rehydration in PBS for 10 min.

For wholemount in situ hybridization chain reaction (HCR), dissected retinas and whole killifish larvae were fixed in 4% paraformaldehyde + 5% sucrose. After fixation, larvae were bleached using 3% H_2_O_2_ and 0.5% KOH in distilled water until pigmentation from the eyes was removed. Whole larvae or adult retinas were dehydrated in methanol and stored at −20°C until use.

### 2.4 in situ Hybridization Chain Reaction

Labeling protocol for in situ HCR was modified from published methods (Choi et al. 2018). The in situ solutions and amplifiers (AlexaFluor‐488, ‐546, ‐594, ‐647) were purchased from Molecular Instruments (https://www.molecularinstruments.com/). Probes against rod transducin (*gnat1*), cone transducin (*gnat2*), and opsin genes (*sws1*, *sws2*, *rh2*, *lws*, *rho*, *opn4a*) were designed using a custom script (Trivedi and Powell, unpublished) and ordered from Life Technologies as DNA oligos. As *lws* and *rh2* had multiple identified genes, probes were made for each identified orthologue and pooled—that is, the *lws* labeling includes probe pairs against all three identified *lws* genes, and *rh2* labeling includes probe pairs made against both *rh2* genes identified. Probes were resuspended to a 100 µM concentration using nuclease‐free water, and all probe pairs for each individual gene were diluted to a final concentration of 1 µM and combined into a stock mix.

*Wholemount:* Tissue was rehydrated in a series of methanol/phosphate buffered saline (PBS) washes for 5 min each as follows: 75% MeOH, 50% MeOH, 25% MeOH, and PBS + 0.1% Tween (PBSTw). Whole larvae were permeabilized with proteinase K digestion for 20 min at room temperature, washed twice with PBSTw, and post‐fixed with 4% PFA for 20 min at room temperature. To remove residual fixative, tissue was washed with PBSTw thrice for 5 min each. Dissected retinas were not proteinase K‐treated. 500 µL of probe hybridization buffer was added to pre‐hybridize the tissue for 30 min at 37°C. Probe solution was prepared by adding 5 µL of each probe set to 500 µL of probe hybridization buffer. The pre‐hybridization solution was removed, and the probe solution added, then tissue incubated overnight at 37°C. The probe solution was then removed, and 500 µL of 37°C wash buffer was added. Tissue was washed four times, 15 min each, at 37°C, then twice for 5 min each with 5xSSCT at room temperature.

Pre‐amplification was performed by adding 500 µL of amplification buffer at room temperature for 30 min. Hairpins were snap‐cooled and mixed with 500 µL amplification buffer at room temperature. Hairpins were prepared by boiling for 90 seconds and snap‐cooling for 30 minutes, then added to amplification buffer. The pre‐amplification buffer was replaced with the hairpin mixture and incubated in the dark at room temperature overnight. Excess hairpins were removed by washing with 5xSSCT at room temperature. Larvae were transferred into 60% glycerol/PBS, and retinas were dissected using sharpened tungsten needles. Retinas were flatmounted for imaging.

*On slides:* Hydrophobic pens were used to draw a border around the tissue, and the tissue was permeabilized for 10 min with proteinase K treatment, followed by two PBSTw rinses and 20‐min postfix in 4% PFA. Probe hybridization buffer was applied and incubated for 30 min at 37°C in a humidity chamber. Note that 2 pmol of each probe set was mixed with hybridization buffer and applied to the slide and incubated at 37°C overnight. 37°C probe wash buffer was used to remove excess probe, 3 × 10 min, followed by 2 × 5 min room‐temperature SSCT washes. Note that 2 µL each of H1 and H2 amplifier hairpins were heated to 95°C, snap‐cooled for 30 min, and added to amplification buffer. Amplifiers were applied to the slide and incubated overnight at room temperature in a humidity chamber stored in the dark. Excess hairpins were removed by washing with 5xSSCT (2 × 5 min then 2 × 30‐min washes), then mounting media and a coverslip were added.

### 2.5 Immunofluorescence

Tissues on slides were blocked for 90 min at room temperature with 10% normal goat serum in PBSTw + 1% DMSO. Primary and secondary antibodies were diluted in 2% normal goat serum in PBSTw + 1% DMSO. Primary antibodies were applied to the slides and incubated overnight at 4°C, followed by 3 × 15 min washes in PBS. Slides were incubated in secondary antibodies overnight at 4°C, then washed 3 × 15 min with PBS. Slides were mounted with Fluoromount G (Cat. No. 00‐4958‐02).

*Antibodies:* The mouse monoclonal zpr‐3 antibody (ZIRC, RRID: AB_10013805), which recognizes zebrafish rhodopsin proteins (Hu et al. 2024), and mouse monoclonal 1D4 antibody (Santa Cruz Biotechnology, Cat. No. sc‐57432, RRID: AB_3212350), which recognizes rhodopsin in mammalian cells and red cones in zebrafish (Yin et al. 2012), were used at a 1:200 concentration unless microconjugated to a fluorophore. Goat anti‐mouse AlexaFluor 647 (Cat. No. A‐21235) or 555 (Cat. No. A‐21422) secondary antibodies were used at a 1:1000 concentration. Microconjugation using the ProteinTech FlexAble CoraLite Plus 555 (Cat. No. KFA022) and 647 (Cat. No. KFA023) kits following the kit directions. For each antibody, the reaction volume was doubled, and 3 µL of antibody was used.

### 2.6 Imaging

Images were acquired using a Zeiss 900 Airyscan 2 confocal microscope.

### 2.7 Nearest Neighbor Distribution and Regularity Index Calculations

Images were segmented using the StarDist (https://github.com/stardist/stardist) plug‐in for Napari (0.6.6). After the segmentation masks were generated, centroids were identified using the nfinder (https://github.com/santi‐rodriguez/nfinder/) plug‐in (Moretti et al. 2023). Nearest neighbors were determined using cKDTree and skimage, and the regularity index (RI) was determined using the following formula:

$$\mathrm{RI}=\frac{\bar{d}}{\sigma }$$

### 2.8 Statistical Analysis

Statistics were performed using GraphPad Prism (v 11.0.0 (93)).

## 3 Results

### 3.1 Adult Turquoise Killifish Have a Cone‐Dominant Retina

**FIGURE 1:**
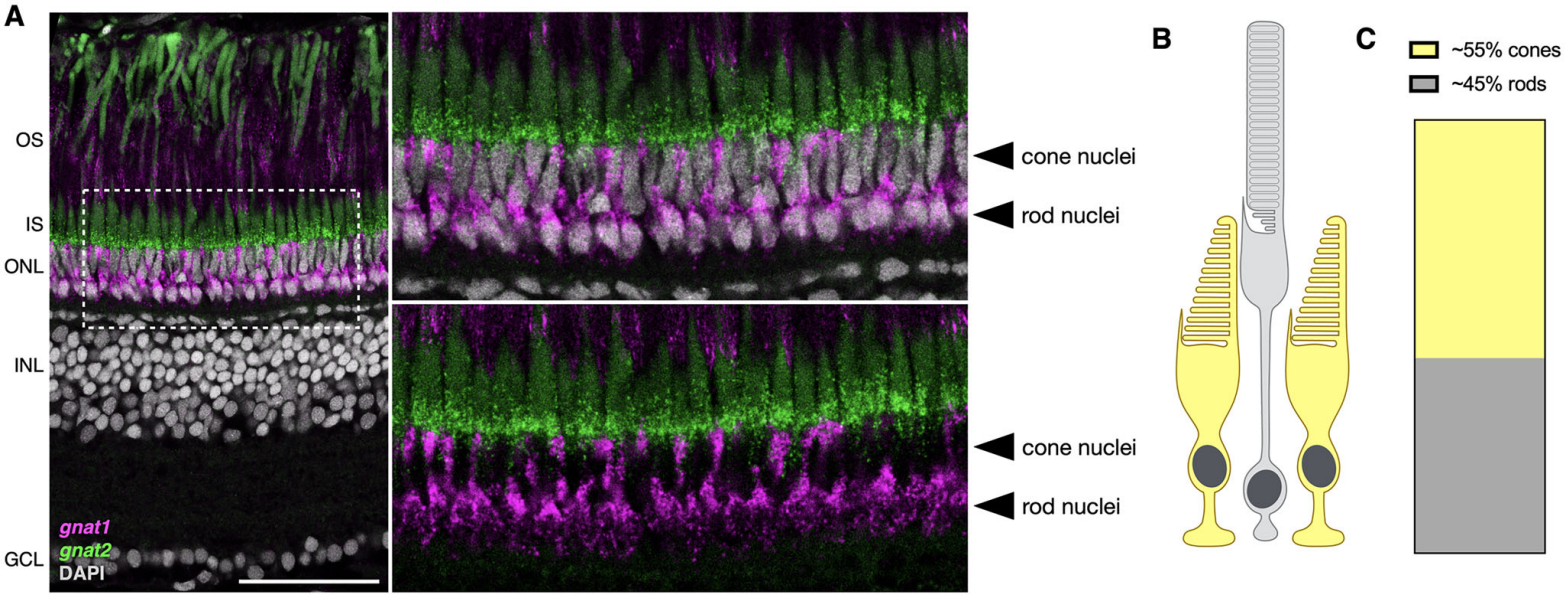
Adult turquoise killifish have a cone‐dominant retina. (A) 6 weeks post hatching killifish retinal cryosection with rods (*gnat1*, magenta) and cones (*gnat2*, green) labeled using in situ hybridization chain reaction. Rod nuclei sit basal to the cone nuclei. (B) Cartoon depicting where the cones (yellow) and rods (grey) are positioned in relation to each other within the outer retina. (C) The adult killifish retina is more than half cone photoreceptors, as determined by counting the rod and cone nuclei within a 150 µm area by randomly sampling retinal sections across *n* = 5 individuals. GCL = ganglion cell layer; INL = inner nuclear layer; IS = inner segments; ONL = outer nuclear layer; OS = outer segments. Scale bars = 25 µm.

Previous studies only determined that adult turquoise killifish had markers for both rods and cones in their retinas—therefore, adult retinas were first investigated for rod:cone ratio. To label rods and cones on tissue sections, in situ HCR was used to visualize cells expressing *gnat1* and *gnat2*, rod‐ and cone‐specific components of the phototransduction pathway, respectively (Figure 1A). Rod nuclei were found to be positioned basally to cone nuclei within the outer nuclear layer (Figure 1A,B), which is typical of vertebrate retinas. The rod nuclei are spherical, while cone nuclei are oblong. Rod and cone nuclei were counted within the outer nuclear layer across randomly sampled 6‐week post hatching young adult turquoise killifish retinal tissue sections to determine the rod:cone ratio. The adult turquoise killifish retinal photoreceptor population is comprised of 55.07 ± 3.25% cones, and is therefore cone‐rich.

### 3.2 Turquoise Killifish Have Four Cone Photoreceptor Subtypes Based on Opsin Gene Expression

**FIGURE 2:**
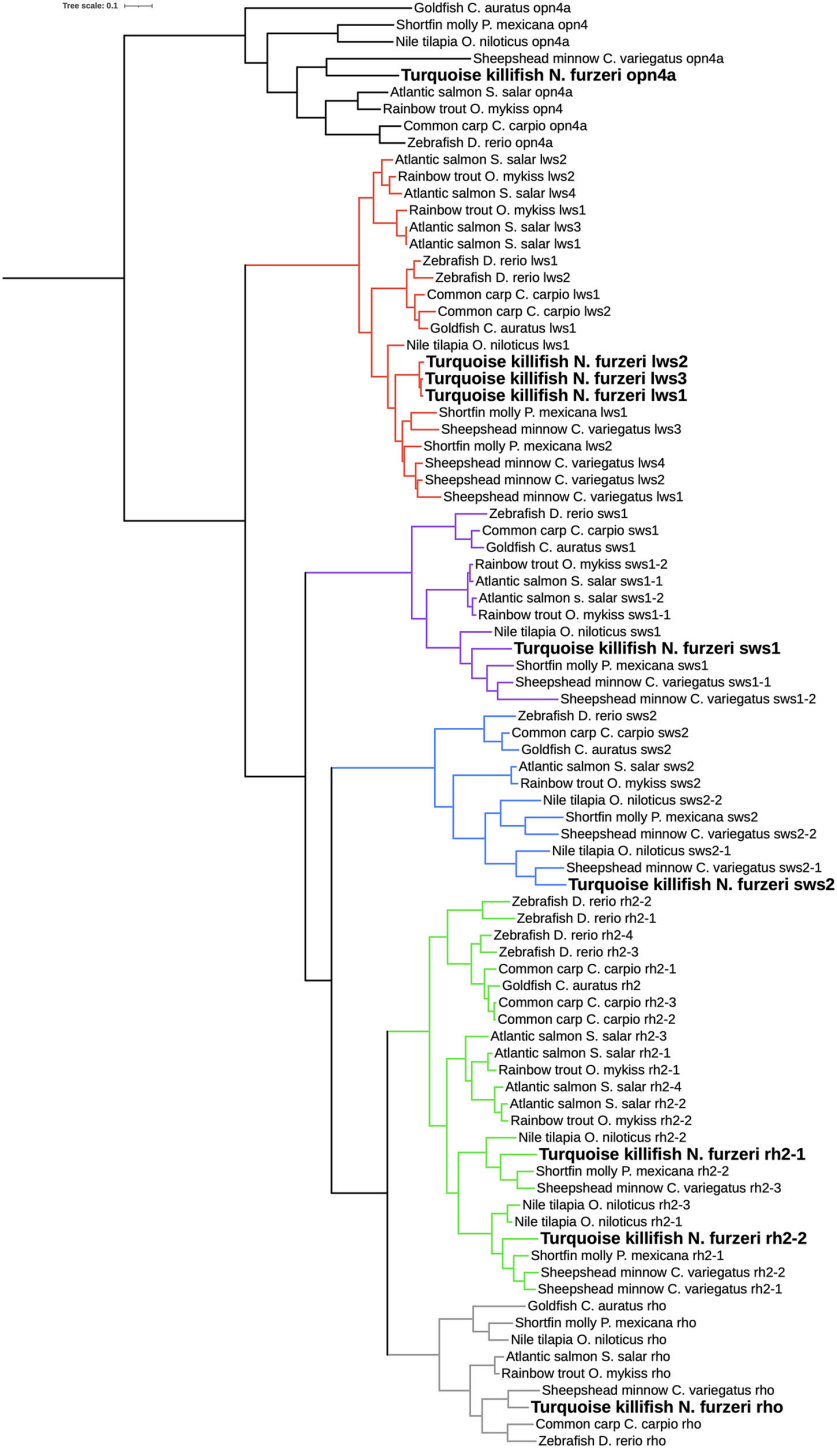
Turquoise killifish have four cone opsin classes and seven cone opsin genes. Maximum likelihood tree of opsin genes (*sws1*, *sws2*, *rh2*, *lws*, and *rho*) in nine teleost fish species (Atlantic salmon, goldfish, sheepshead minnow, shortfin molly, rainbow trout, Nile tilapia, common carp, zebrafish, and turquoise killifish) using melanopsin (*opn4a*) to root the tree. Tree was generated using IQ‐Tree with 1000 bootstrap replicates and visualized using iTOL.

The turquoise killifish genome (NfurGRZ‐RIMD1) was assessed for visual opsin genes to identify the potential photoreceptor types within the turquoise killifish retina. The following potential opsin genes were identified: one rhodopsin (*rho*) gene, one UV opsin (*sws1*) gene, one blue opsin (*sws2*) gene, two green opsin (*rh2*) genes, and three red opsin (*lws*) genes. Opsin genes from zebrafish (*D. rerio*), goldfish (*C. auratus*), common carp (*C. carpio*), Atlantic salmon (*S. salar*), rainbow trout (*O. mykiss*), Nile tilapia (*O. niloticus*), shortfin molly (*P. mexicana*), and sheepshead minnow (*C. variegatus*) were used to assess whether the identified turquoise killifish genes were indeed visual opsins and grouped with the same opsin genes from other species, thereby being appropriately annotated. Further, this assessment shows the relatedness of the identified putative opsin genes with other teleosts opsin genes. Melanopsin (*opn4a*), as a nonvisual opsin, was used to root the tree. The relationships inferred by the tree indicate that the opsin orthologues identified in the turquoise killifish genome are appropriately annotated; all opsin genes identified in the turquoise killifish grouped with the same opsin genes across other teleost species, demonstrating sequence homology (Figure 2). Turquoise killifish opsin gene sequences were most similar to opsin genes from sheepshead minnow, a non‐annual killifish species, and shortfin molly (Figure 2).

**FIGURE 3:**
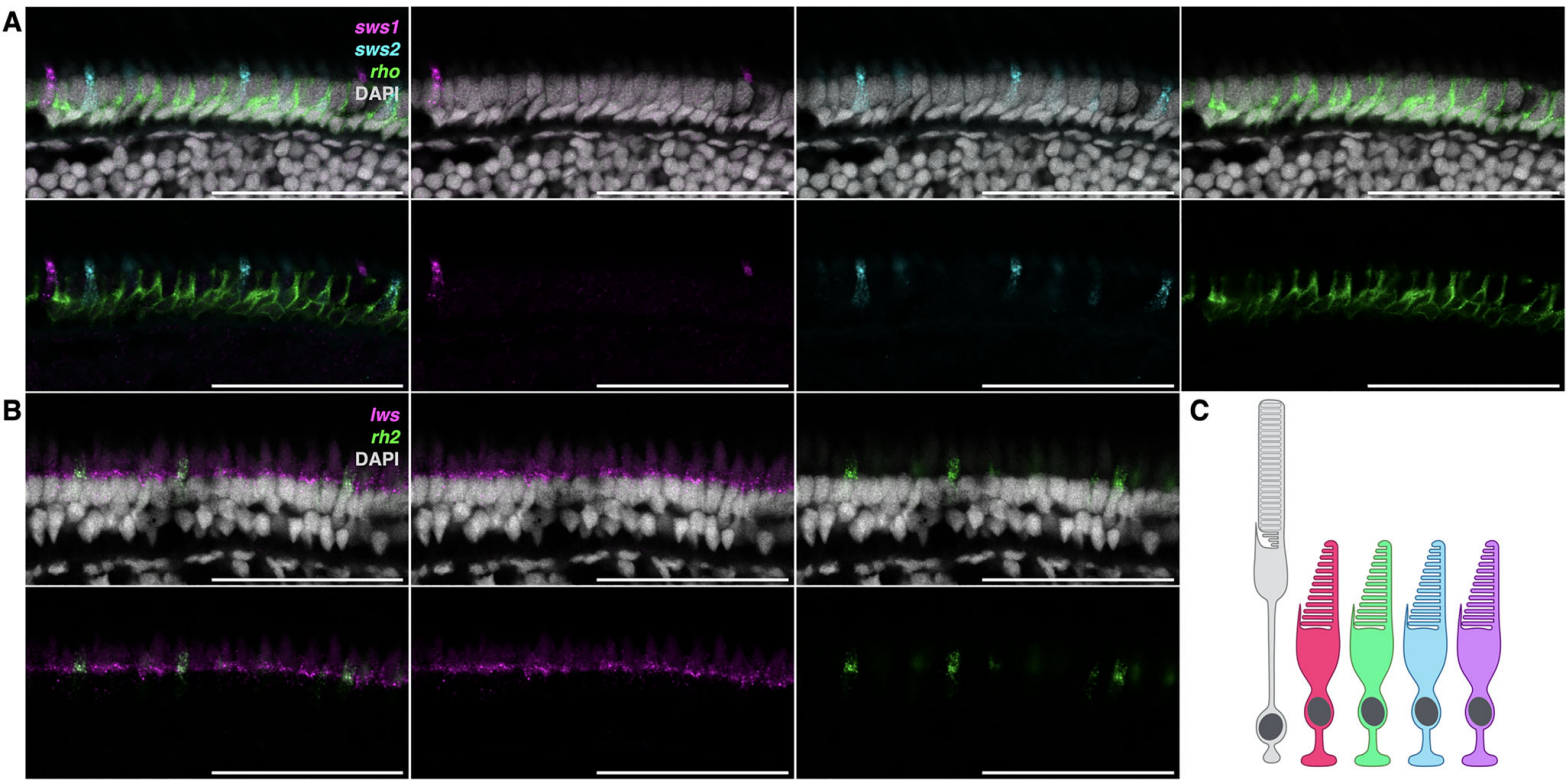
Rhodopsin and four cone opsin classes are expressed in the adult turquoise killifish retina. (A) In situ hybridization chain reaction labeling for UV opsin (*sws1*, magenta), blue opsin (*sws2*, cyan), and rhodopsin (*rho*, green) with (top) and without (bottom) DAPI. (B) In situ hybridization chain reaction labeling for green (*rh2*, green) and red (*lws*, magenta) cone opsins with (top) and without (bottom) DAPI. (C) The turquoise killifish have rods as well as four cone subtypes, expressing red, green, blue, and UV opsins. Scale bars = 25 µm.

To determine whether the identified opsin gene classes are expressed in the adult turquoise killifish retina, in situ HCR was used. This labeling revealed that adult killifish photoreceptors express *sws1*, *sws2*, *rh2*, *lws*, and *rho* in distinct cells (Figure 3). The most visually widespread cone opsin class appeared to be *lws*, which had largely uniform expression across the photoreceptor layer, while *sws1*, *sws2*, and *rh2* had comparatively sparse expression.

### 3.3 Turquoise Killifish Have Double Cones With Units That Can Express the Same or Different Opsins

The retina was investigated for double cones using antibody labeling. In zebrafish, double cones are comprised of a principal red and accessory green cone unit (Larison and Bremiller 1990; Vihtelic et al. 1999b). The zpr‐3 antibody (ZIRC, RRID: AB_10013805) labels both rod and green cone rhodopsin (Hu et al. 2024), while the 1D4 antibody (Santa Cruz Biotechnology, Cat. No. sc‐57432, RRID: AB_3212350) labels red cones (Yin et al. 2012) in zebrafish, allowing for the double cone subunits to be distinguished. These antibodies were used on the turquoise killifish retina to investigate red/green double cones.

**FIGURE 4:**
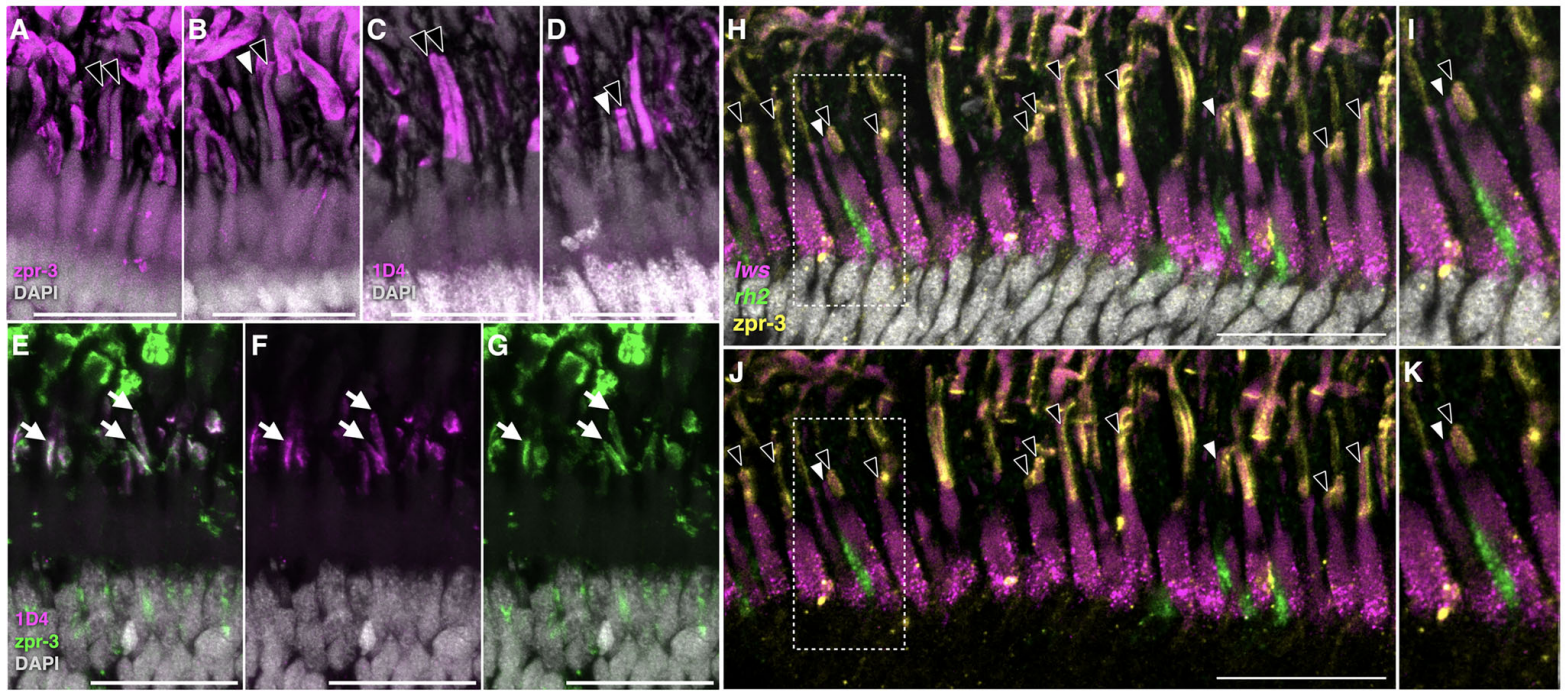
Double cones expressing the same or different opsins are present in the adult killifish. Physically fused double cones with subunits that are indistinguishable based on morphology are observed when labeling with zpr‐3 (A, B) and 1D4 (C, D) antibodies. Double cones can have units that both express the same (A, C) or different (B, D) opsin proteins. Black arrowheads indicate antibody‐labeled double cone units and white arrowheads indicate unlabeled double cone units. Immunolabeling with both zpr‐3 (green) and 1D4 (magenta) (E–G) shows that the antibodies have overlapping expression in cone outer segments (arrows). (H–K) in situ hybridization chain reaction for green (rh2, green) and red (lws, magenta) opsins followed by immunolabeling with zpr‐3 (yellow), with (K, I) and without (J, K) DAPI. Zpr‐3 outer segment labeling is observed on lws‐expressing red cones (black arrowheads) and is absent from rh2‐expressing green cones (white arrowheads). Scale bars = 25 µm.

Retinas were found to have double cones with units that were indistinguishable based on morphology (Figure 4A–D). Interestingly, when retinas were labeled with zpr‐3 or 1D4, these double cones could contain either two cells that were simultaneously labeled, presumably expressing the same opsin class, or two cells that did not simultaneously label, presumably expressing different opsin classes. Double cone units most frequently appeared to be expressing the same opsin. If zpr‐3 were labeling green cones (as well as rods) while 1D4 labeled red cones, as in zebrafish, it is expected that there would be double cones without any outer segment labeling when using zpr‐3, reflecting double cones whose units were both expressing 1D4‐recognized red opsin—however, that was not observed. To determine whether the similarity of the zpr‐3 and 1D4 labeling in double cones was due to both antibodies labeling the same cone subtype in turquoise killifish, tissue was labeled with zpr‐3 and 1D4 simultaneously. Indeed, cone labeling with these antibodies overlapped, while zpr‐3 additionally labeled rods (Figure 4E–G). To determine whether zpr‐3 and 1D4 antibodies were recognizing green or red cones, in situ HCR was performed for *rh2* and *lws* with subsequent antibody labeling with zpr‐3. Cells that were positive for *rh2* labeling did not have outer segment labeling with zpr‐3, while *lws*‐positive cells had zpr‐3 outer segment labeling (Figure 4H–K). Therefore, zpr‐3 and 1D4 antibodies label red cones in turquoise killifish. In sum, adult turquoise killifish have double cones without obvious principal or accessory cone morphologies using semi‐super‐resolution confocal microscopy. Double cones can contain one red and one green opsin‐expressing cell or two red opsin‐expressing cells.

### 3.4 Adult Turquoise Killifish Have a Square Mosaic Where Red Cones Are the Predominant Cone Subtype

**FIGURE 5:**
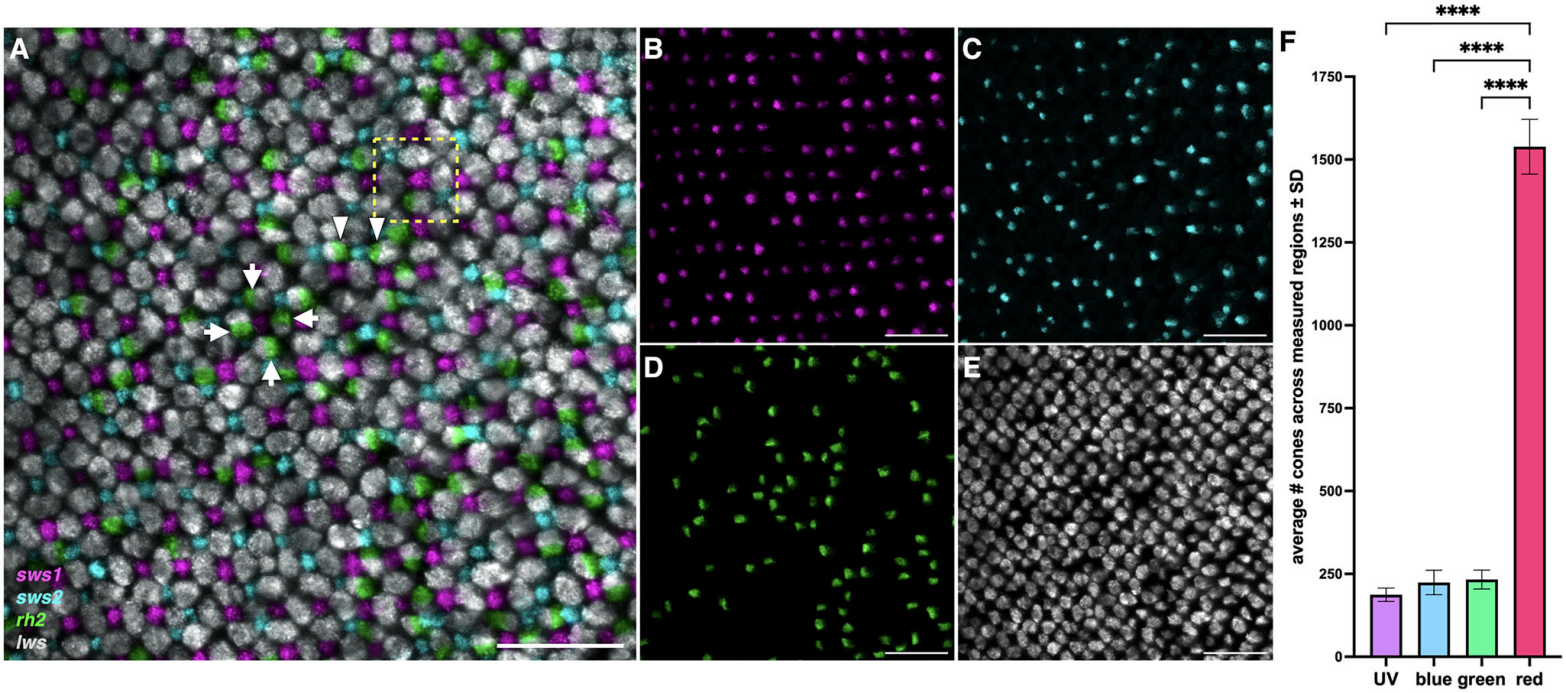
Cones in the adult retina are organized in a red cone‐dominant square mosaic. (A‐E) Retinal flatmount of 6 weeks post hatching adult killifish retina labeled for UV (*sws1*, magenta), blue (*sws2*, cyan), green (*rh2*, green), and red (*lws*, grey) cones using in situ hybridization chain reaction. Killifish have a square mosaic with green and red cones forming squares across the retina (yellow box). Green cones within a square unit alternate with red cones (arrows), while in corners where two square units meet, they are apposed (arrowheads). (F) All UV, blue, green, and red cones were counted in 100 µm × 100 µm boxes taken in the ventral, nasal, dorsal, and temporal retina. The average number of cones in the adult killifish is shown, ± standard deviation. *n* = 4. One‐way ANOVA with Tukey's post hoc multiple comparisons test, ****p < 0.0001. Scale bars = 25 µm.

**FIGURE 6:**
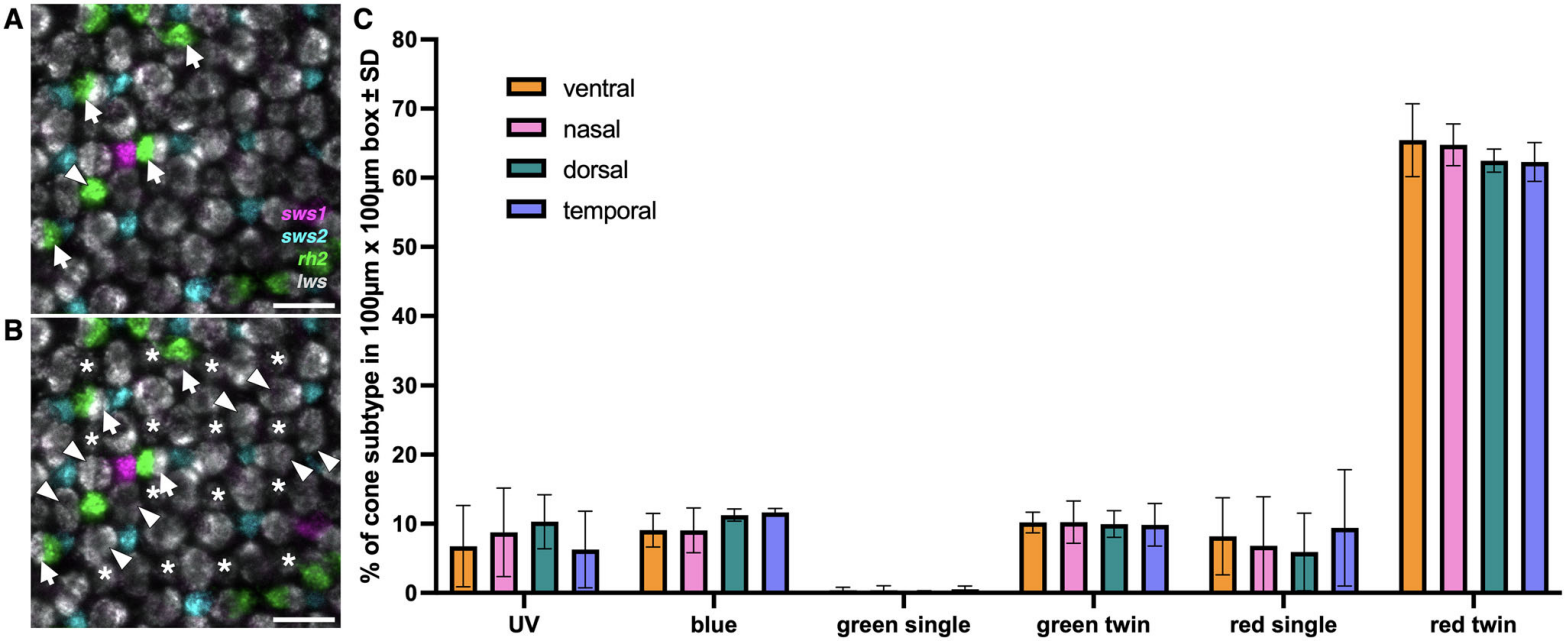
Cone subtype abundance is evenly distributed across the retina. (A) Adult killifish have green twin (arrows) and rare green single (arrowhead) cones, as observed when labeling UV (*sws1*, magenta), blue (*sws2*, cyan), green (*rh2*, green), and red (*lws*, grey) cones by in situ hybridization chain reaction. (B) Red cones can be a subunit of a cone doublet with either another red cone (arrowheads) or a green cone (arrows) or exist as single cones (asterisks). (C) Graph showing the average number of each cone subtype across the ventral, nasal, dorsal, and temporal retina, ± standard deviation. *n* = 4. Two‐way ANOVA with Tukey's post hoc multiple comparisons test. Scale bars = 10 µm.

To assess the photoreceptor mosaic organization, cone subtypes were labeled with in situ HCR against *sws1*, *sws2*, *rh2*, and *lws* transcripts. Adult turquoise killifish were found to have a square mosaic, where double cones form square units around central single cones (Figure 5). Additionally, single cones can sit at the corners of the squares. Interestingly, and unlike adult zebrafish, adult turquoise killifish have single red cones and rare single green cones (Figure 6A,B). Single cones within square centers were UV, blue, red, or—very rarely—green in identity, whereas single cones at square corners were either UV or blue. When double cones contained green and red cone subunits within their square formation, the green cones did not face each other within the square, and as a result, green cones from neighboring squares faced each other where the corners of the squares meet (Figure 5A). To determine the cone subtype abundance, cone subtypes were counted within boxes in the dorsal, nasal, ventral, and temporal retinas. These analyses confirmed that red cones are the predominant cone subtype in the adult turquoise killifish retina (Figure 5F), making up 70.54 ± 1.59% of the cone population, while green cones were 10.66 ± 0.68%, blue cones were 10.24 ± 1.26%, and UV cones were 8.56 ± 0.32%. When comparing the abundance of each cone subtype across the investigated retinal areas to determine whether there was regionalization of photoreceptor concentration, there was no significant difference between retinal regions (Figure 6C).

### 3.5 The Turquoise Killifish Square Mosaic Is Ordered but Lacks Cone Subtype Regularity

**FIGURE 7:**
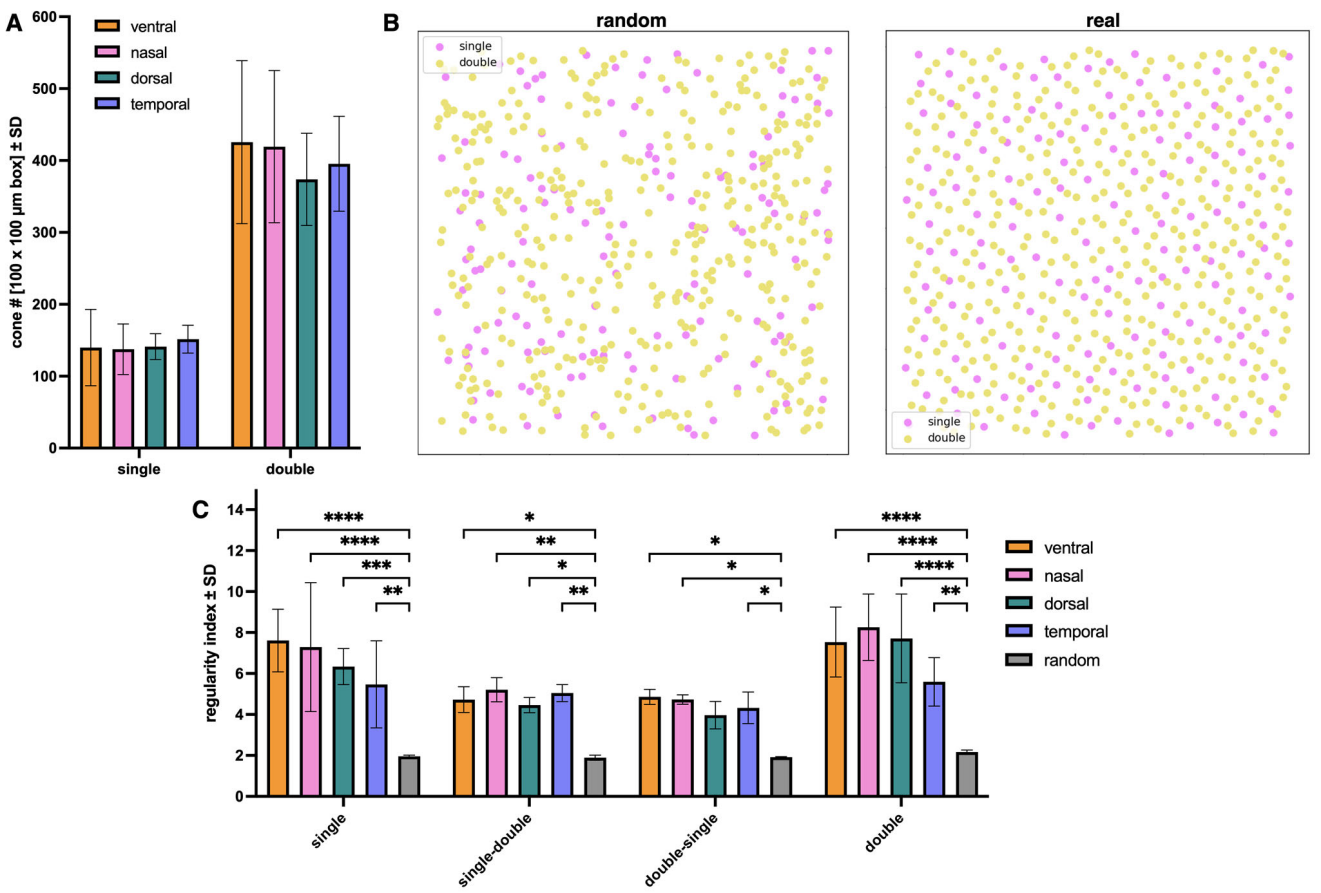
Single and double cones in the adult retina have spatial regularity. (A) Number of cones counted ± standard deviation in the ventral, nasal, dorsal, and temporal retina. The number of single cones did not differ across retinal regions, nor did the number of double cones. (B) Centroids for a randomly seeded simulation (left) and real mosaic (right) of single (pink) and double (yellow) cones. Given that the number of cones forming single or double cone units did not differ by retinal region, the average value of each was used in the random simulation. (C) Regularity index values ± standard deviation for homotypic and heterotypic single and double cone pairs in the ventral, nasal, dorsal, temporal, and random simulation. Single and double cones across the retina have significantly higher regularity indexes in both homotypic and heterotypic pairings than the random simulation. *n* = 4. Two‐way ANOVA with Tukey's post hoc multiple comparisons test, *p < 0.05; **p < 0.01; ***p < 0.001; ****p < 0.0001.

Regularity index (RI) was employed to measure the geometric order of the mosaic (Figure 7). RI is calculated by dividing the average nearest neighbor distance by the standard deviation. Cone type—single and double—was assessed to determine whether the mosaic overall had regularity, independent of cone subtype identity, by comparison to a randomly seeded simulation. Of note, the number of cones existing as single or double cone units was not statistically different between retinal regions (Figure 7A), and therefore, the average cone number was used to generate the randomly seeded simulation (Figure 7B). When comparing homotypic (single to single, double to double) and heterotypic (single to double, double to single) cone pairs across retinal regions, the RIs were significantly higher than the random simulation. This highlights that the mosaic has significant spatial regularity overall.

**FIGURE 8:**
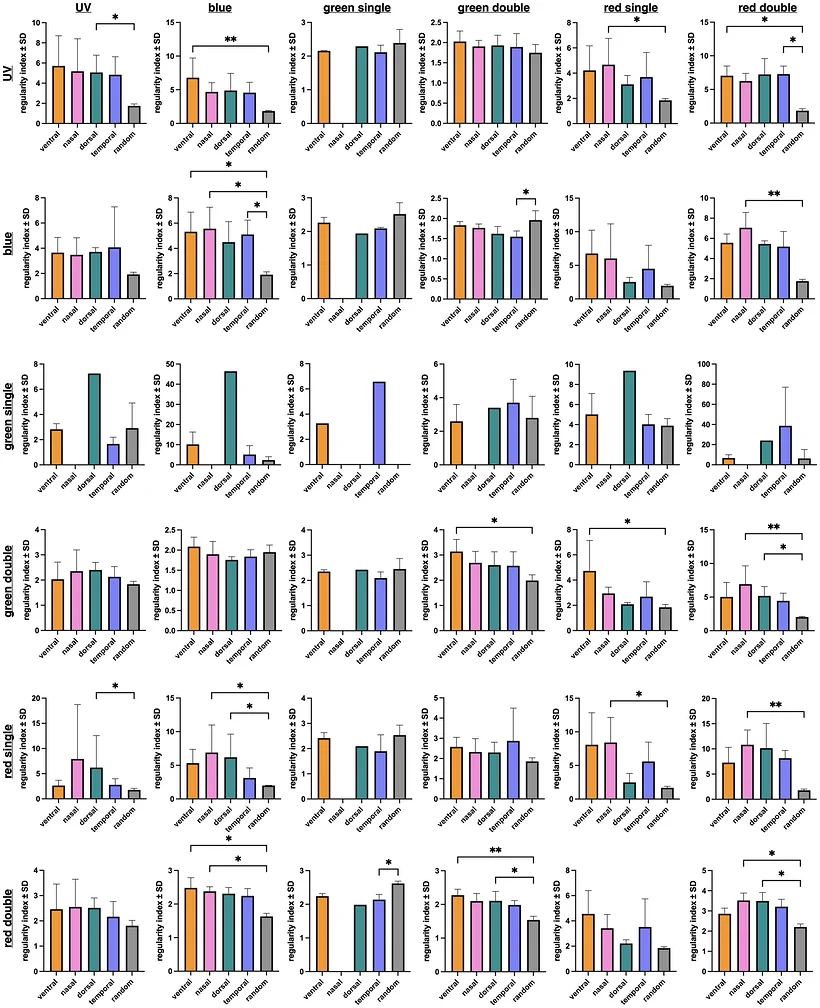
Regularity index values for cone homotypic and heterotypic pairs across ventral, nasal, dorsal, and temporal retina, compared to random simulations. n=4 for nasal, dorsal, ventral; n=5 for temporal; n=3 randomly seeded simulations. Kruskal–Wallis multiple comparisons test, *p < 0.05; **p < 0.01.

While the mosaic overall has regularity, the cone subtypes within the mosaic may not have spatial co‐regularities. The RIs for homotypic (UV to UV, blue to blue, etc.) and heterotypic (UV to blue, UV to green double cone, etc.) pairs were calculated for every cone subtype combination across retinal regions and compared to random simulations (Figure 8, Tables 1, 2, 3, 4). The random simulation used the average number of cones per subtype, as there was no significant difference in subtype abundance across retinal areas (Figure 6). While some subtype combinations did have statistically significantly higher or lower RIs compared to the random simulation in certain retinal regions, this was not consistent across retina regions (Figure 8, Tables 1, 2, 3, 4). Further, the majority of subtype combinations failed to reach statistical significance. Thus, the regularity of the cone subtypes was not significantly different from random across the retina, indicating that the cone subtypes have stochasticity in their orientation within the ordered mosaic.

**TABLE 1:** Regularity index calculated for all cone subtype combinations for the adult ventral retina. Yellow cells indicate relationships that are significantly higher than the randomly seeded simulation, and therefore are more regularly oriented.

	UV	Blue	Green S	Green DC	Red S	Red DC
UV	5.71 ± 2.99	6.79 ± 2.9**	2.16 ± 0.01	2.03 ± 0.26	4.22 ± 1.95	7.05 ± 1.43*
Blue	3.65 ± 1.21	5.32 ± 1.56*	2.26 ± 0.16	1.83 ± 0.09	6.76 ± 3.47	5.56 ± 0.87
Green S	2.82 ± 0.45	10.16 ± 6.11	—	2.59 ± 1.00	5.02 ± 2.08	6.72 ± 3.26
Green DC	2.03 ± 0.68	2.09 ± 0.23	2.35 ± 0.07	3.14 ± 0.48*	4.23 ± 2.42*	5.03 ± 2.15
Red S	2.64 ± 1.05	5.31 ± 2.06	2.41 ± 0.22	2.58 ± 0.47	8.08 ± 4.75	7.28 ± 3.02
Red DC	2.46 ± 1.00	2.48 ± 0.30*	2.24 ± 0.08	2.27 ± 0.18**	4.56 ± 1.83	2.86 ± 0.28

*p < 0.05; **p < 0.01.

**TABLE 2:** Regularity index calculated for all cone subtype combinations for the adult nasal retina. Yellow cells indicate relationships that are significantly higher than the randomly seeded simulation, and therefore are more regularly oriented.

	UV	Blue	Green S	Green DC	Red S	Red DC
UV	5.18 ± 3.23	4.68 ± 1.37	—	1.90 ± 0.15	4.67 ± 2.08*	6.24 ± 1.15
Blue	3.48 ± 1.35	5.57 ± 1.70	—	1.77 ± 0.09	6.02 ± 5.15	7.05 ± 1.52**
Green S	—	—	—	—	—	—
Green DC	2.35 ± 0.85	1.89 ± 0.32	—	2.69 ± 0.45	2.94 ± 0.50	6.92 ± 2.72**
Red S	7.93 ± 10.79	6.90 ± 4.09*	—	2.32 ± 0.66	8.41 ± 3.74	10.82 ± 2.94**
Red DC	2.55 ± 1.10	2.38 ± 0.13*	—	2.10 ± 0.22	3.41 ± 1.10	3.52 ± 0.37*

*p < 0.05; **p < 0.01.

**TABLE 3:** Regularity index calculated for all cone subtype combinations for the adult dorsal retina. Yellow cells indicate relationships that are significantly higher than the randomly seeded simulation, and therefore are more regularly oriented.

	UV	Blue	Green S	Green DC	Red S	Red DC
UV	5.06 ± 1.72*	4.88 ± 2.55	—	1.93 ± 0.25	3.11 ± 0.70	7.23 ± 2.35
Blue	3.71 ± 0.34	4.49 ± 1.62	—	1.62 ± 0.18	2.53 ± 0.67	5.45 ± 0.30
Green S	—	—	—	—	—	—
Green DC	2.40 ± 0.30	1.76 ± 0.08	—	2.60 ± 0.53	2.08 ± 0.14	5.19 ± 1.38*
Red S	6.20 ± 6.35*	6.19 ± 3.44*	—	2.30 ± 0.51	2.48 ± 1.32	10.14 ± 4.89
Red DC	2.51 ± 0.39	2.31 ± 0.18	—	2.11 ± 0.28*	2.21 ± 0.29	3.49 ± 0.42*

*p < 0.05.

**TABLE 4:** Regularity index calculated for all cone subtype combinations for the adult temporal retina. Yellow cells indicate relationships that are significantly higher than the randomly seeded simulation, and therefore are more regularly oriented. Orange cells indicate relationships that are significantly lower than the random simulation, clustered in distribution. All other cells are not significantly different from the randomly seeded simulation.

	UV	Blue	Green S	Green DC	Red S	Red DC
UV	4.84 ± 1.78	4.56 ± 1.54	2.11 ± 0.21	1.89 ± 0.33	3.69 ± 1.95	7.28 ± 1.18*
Blue	4.07 ± 3.21	5.11 ± 1.14*	2.09 ± 0.03	1.55 ± 0.14*	4.50 ± 3.48	5.18 ± 1.50
Green S	1.66 ± 0.53	5.11 ± 4.41	—	3.70 ± 1.39	4.03 ± 0.98	38.72 ± 38.32
Green DC	2.13 ± 0.41	1.84 ± 0.17	2.09 ± 0.24	2.57 ± 0.55	2.69 ± 1.17	4.44 ± 1.14
Red S	2.79 ± 1.20	3.12 ± 1.49	1.90 ± 0.65	2.87 ± 1.62	5.60 ± 2.86	8.16 ± 1.51
Red DC	2.16 ± 0.60	2.24 ± 0.22	2.14 ± 0.16*	1.98 ± 0.13	3.52 ± 2.23	3.21 ± 0.36

*p < 0.05.

### 3.6 Turquoise Killifish Larvae Retinas Are More Cone Enriched Than Adult Retinas

**FIGURE 9:**
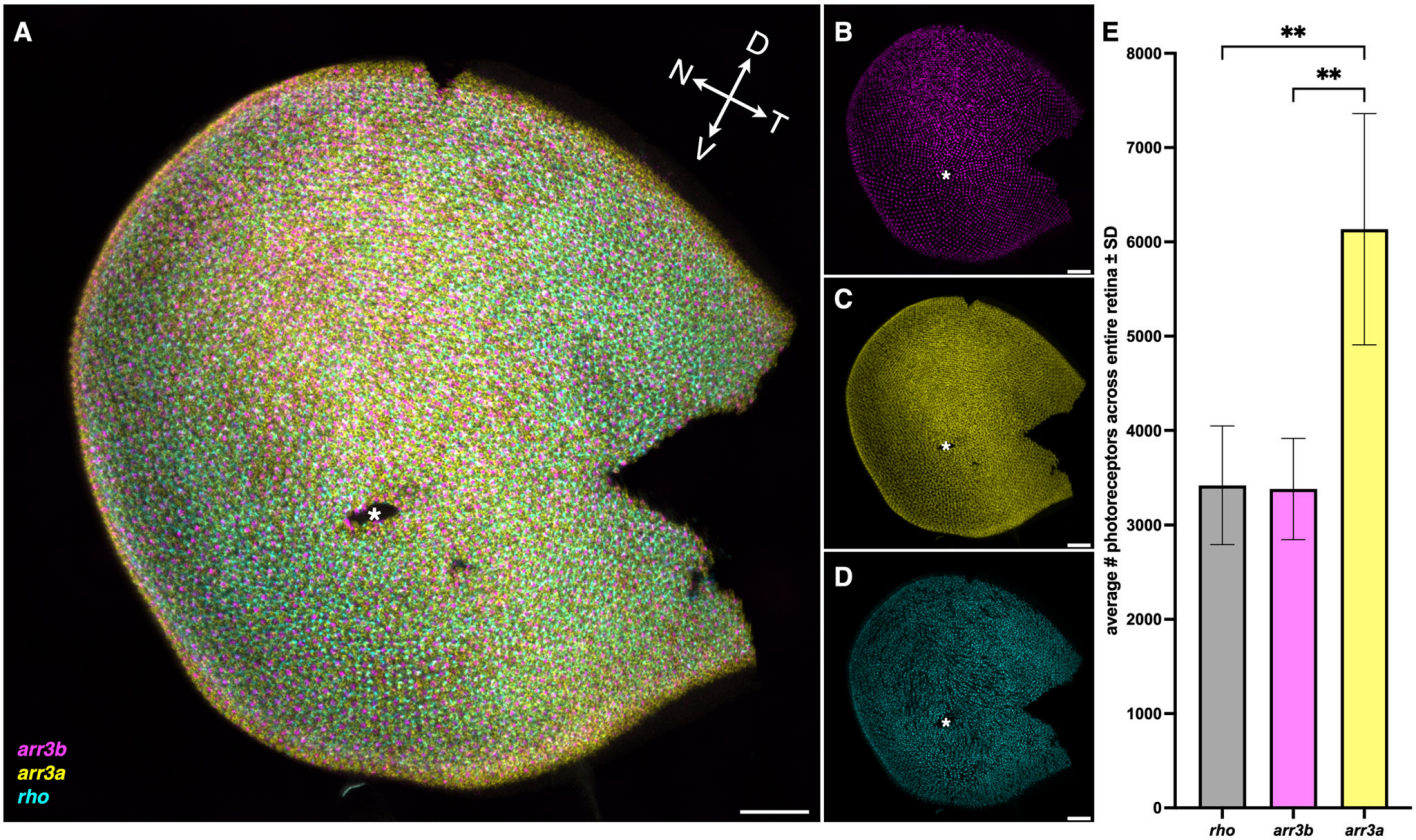
Larval killifish have a cone‐dominant retina. (A) Flatmount of 1‐day post hatching larval killifish retina with short‐wavelength cones labeled with *arr3b* (magenta, B), long‐wavelength cones labeled with *arr3a* (yellow, C), and rods labeled with *rho* (cyan, D) by in situ hybridization chain reaction. On the fluorescent micrographs, *optic nerve head. (E) Average number of *rho* (grey), *arr3b* (magenta), and *arr3a* (yellow)‐positive photoreceptor cells across *n* = 4 larval retinas ± standard deviation. *arr3a*‐positive cones are significantly more abundant than arr3b‐positive cones or rho‐positive rods. One‐way ANOVA with Tukey's post hoc multiple comparisons test, **p < 0.01. Scale bars = 50 µm.

Larval fish generally have different photoreceptor proportions than adults, reflecting distinct visual niches and behaviors between the life stages. To determine the ratio of rods and cones in the larval retina, 1‐day post hatching turquoise killifish retinas were labeled using in situ HCR with probes against rods (*rho*) and cones (*arr3b* and *arr3a*) (Figure 9A–D). In fishes, Arr3a is typically expressed in green and red cones, while Arr3b is expressed in UV and blue cones. The photoreceptor population in larval turquoise killifish retinas was found to be 26.45 ± 1.53% rod photoreceptors; therefore, their retinal photoreceptor population is nearly 75% cone photoreceptors, more cone‐enriched than the adult retina (Figure 1).

**FIGURE 10:**
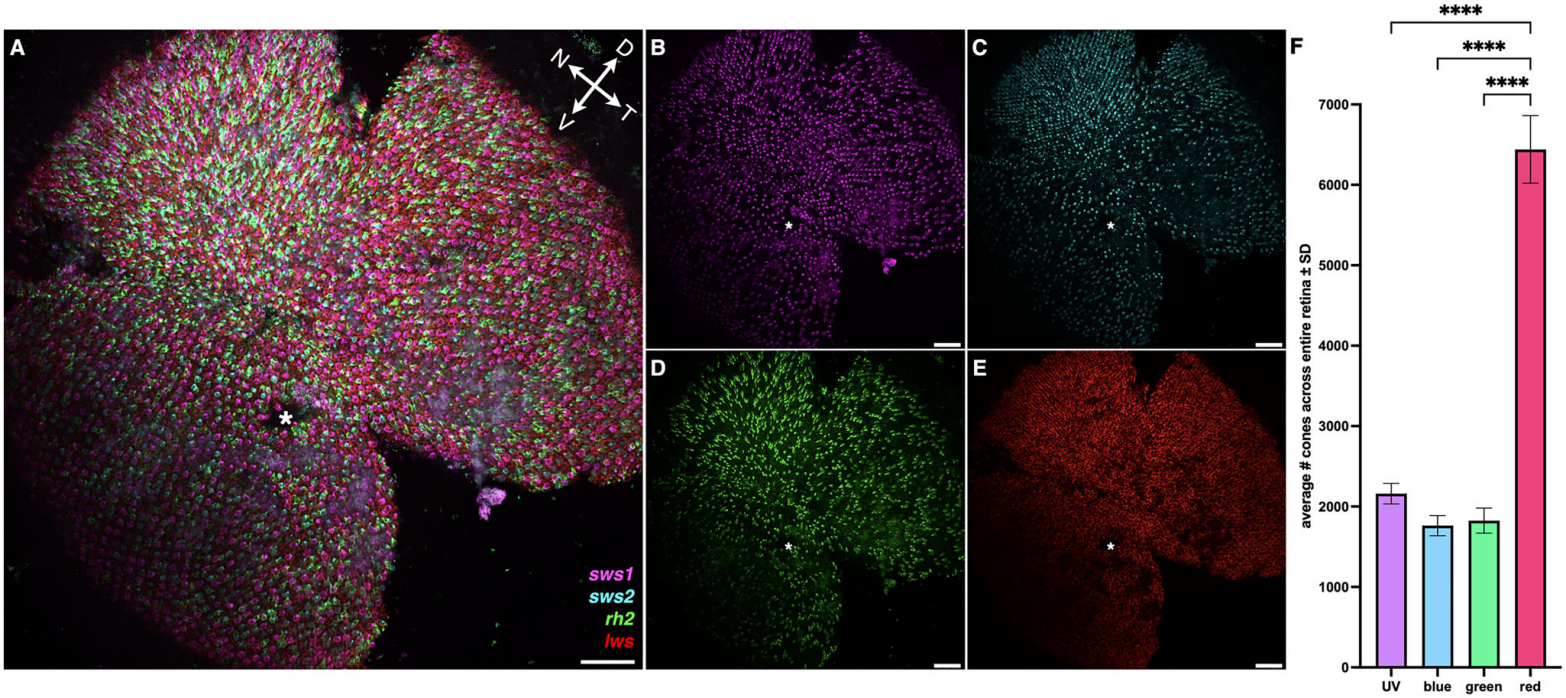
Red cones are the predominant cone subtype in the larval retina. (A) Flatmount of a whole larval killifish retina, with UV (*sws1*, magenta; B), blue (*sws2*, cyan; C), green (*rh2*, green; D), and red (*lws*, red; E) cones labeled by in situ hybridization chain reaction. *Optic nerve head. (F) The average number of cones per subtype across the entire larval killifish retina is shown, ± standard deviation. *n* = 5 for UV, blue, and green; *n* = 4 for red. One‐way ANOVA with Tukey's post hoc multiple comparisons test, ****p < 0.0001. Scale bars = 50 µm.

Next, larval killifish retinas were investigated for cone subtype proportions by labeling with in situ HCR to visualize the full complement of cone photoreceptors. Larval killifish retinas had staining for all cone subtype classes—UV, blue, green, and red—observed in the adults (Figure 10A–E). Cones were counted across the entire retina to determine the proportions of the cone subtypes within the entire cone population (Figure 10F). The larval killifish retina is red cone‐dominant, with *lws*‐expressing cones comprising 52.43 ± 1.12% of the cone population; UV, blue, and green cones were 17.74 ± 0.65%, 14.66 ± 0.61%, and 15.16 ± 0.47% of the cone population, respectively.

### 3.7 The Larval Mosaic Has Regionalization, With a Dorsonasal Region of Increased Blue and UV Cone Density

**FIGURE 11:**
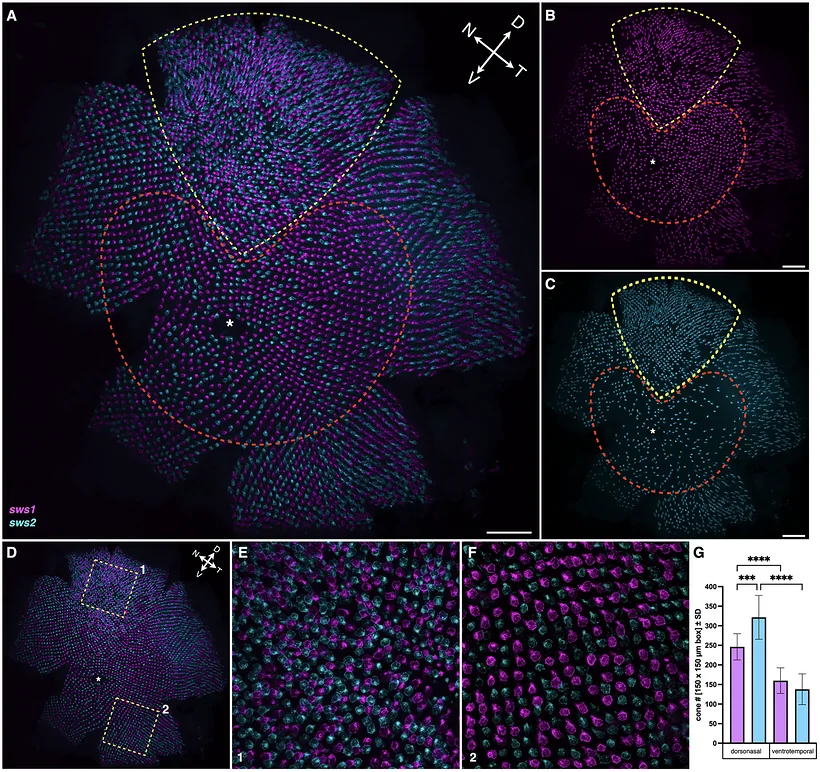
Larval killifish retinas have a dorsonasal region of increased blue cone density. (A) Flatmount of a larval killifish retina with UV (*sws1*, magenta) and blue (*sws2*, cyan) opsins labeled by in situ hybridization chain reaction. There is a dorsonasal region with an apparent increase in blue cone density (yellow dashed line) as well as a central retinal region with sparse blue cones (red dashed line). (B) UV cones; (C) blue cones. *Optic nerve head. (D) Boxes indicate regions that were quantified to determine differential cone density, with (1) corresponding to the dorsonasal region and (2) corresponding to the ventrotemporal region. *Optic nerve. (E) Dorsonasal region with a visual increase in cone density. UV and blue cones are visually crowded and not evenly spaced in this region. (F) Ventrotemporal region with evenly spaced blue and UV cones. (G) Quantification of UV and blue cones within 150 µm × 150 µm boxes. There is a significant increase in UV and blue cone density within the dorsonasal region compared to the ventrotemporal region, with blue cones being especially abundant. *n* = 6. ANOVA with Tukey's post hoc multiple comparisons test, ***p < 0.001, ****p < 0.0001. Scale bars = 50 µm.

Retinal regionalization has been observed in larval zebrafish (Yoshimatsu et al. 2020), and turquoise killifish larval retinas were assessed for similar signs of regionalization. The larval retina had apparent regionalization of blue and UV cones—the central retina was blue cone sparse compared to the peripheral retina, and the dorsonasal region had visually increased blue cone density (Figure 11A–C). To characterize this apparent increase in blue cone abundance in the dorsonasal region, UV and blue cones were counted within the dorsonasal region as well as the ventrotemporal region directly across (Figure 11D–G). The dorsonasal region had significantly more UV and blue cones compared to the ventrotemporal region (Figure 11G). Blue cones were >2× more abundant in the dorsonasal region than in the ventrotemporal region, while UV cones were 1.5× more abundant. Blue cones were significantly more abundant than UV cones in the dorsonasal region, demonstrating the blue cone enrichment.

**FIGURE 12:**
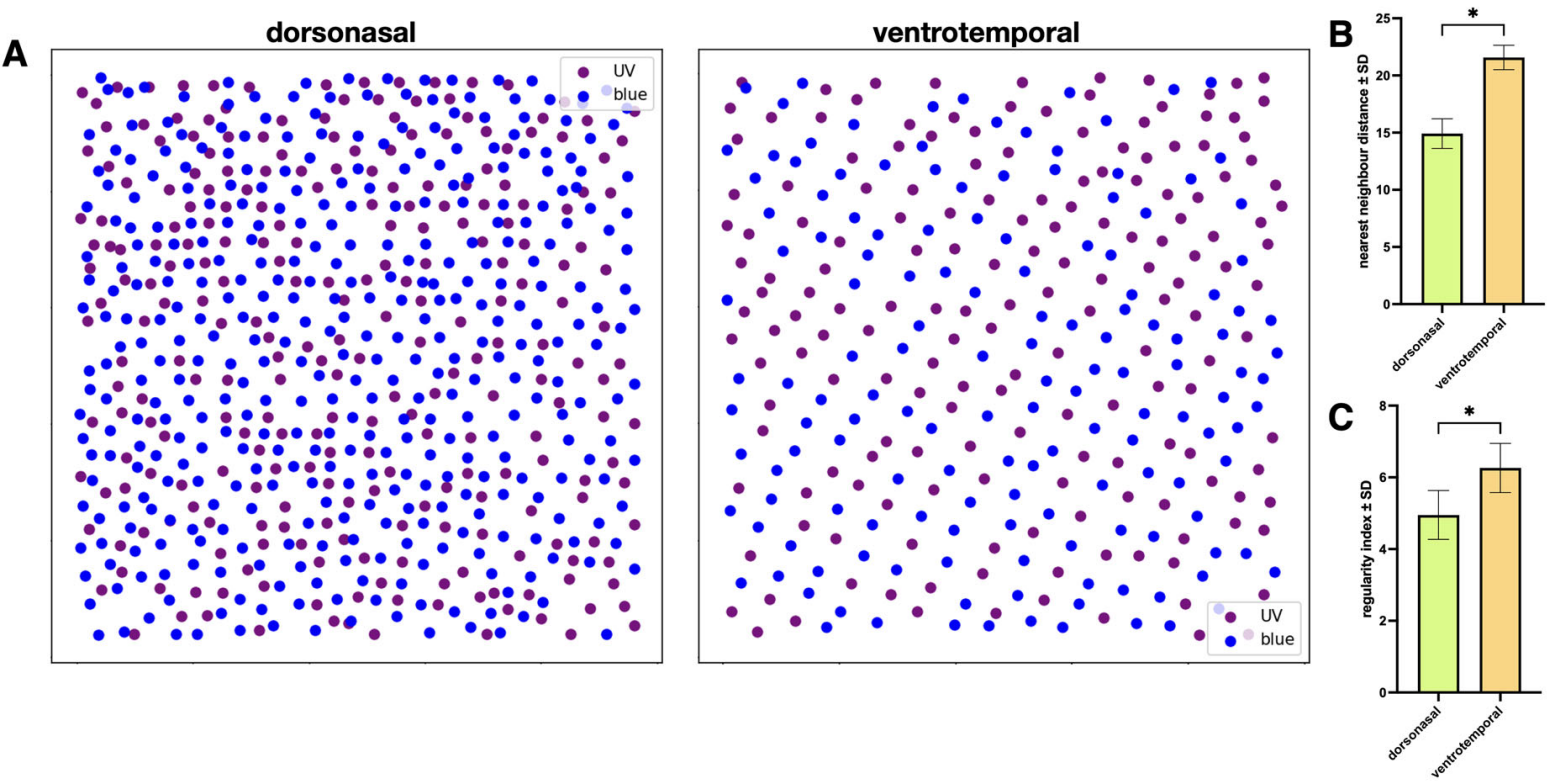
UV and blue cone population in the ventrotemporal retina shows more regularity than in the dorsonasal retina. (A) Centroids of blue and UV cones observed in the dorsonasal (left) and ventrotemporal (right) larval turquoise killifish retina. (B) The ventrotemporal region has significantly more space between the UV and blue cones than the dorsonasal region. (C) The ventrotemporal area has significantly higher regularity than the dorsonasal area. *t*‐test, *p < 0.05.

The UV and blue cones appeared uniformly spaced in the ventrotemporal region and clustered in the dorsonasal area (Figure 12A). The regularity of the UV and blue cones was investigated by treating the UV and blue cones as a single population—the short‐wavelength cone population—and comparing the nearest neighbor distance and RI between the regions. The nearest neighbor distance was significantly lower for the short‐wavelength cone population in the dorsonasal region, indicating that the cells are more packed together in this area (Figure 12B). Further, the RI for the dorsonasal region was significantly lower than the ventrotemporal region (Figure 12C). This demonstrates that the ventrotemporal larval retina has more regularity in the spatial orientation of the short‐wavelength cone population than the dorsonasal region.

## 4 Discussion

**FIGURE 13:**
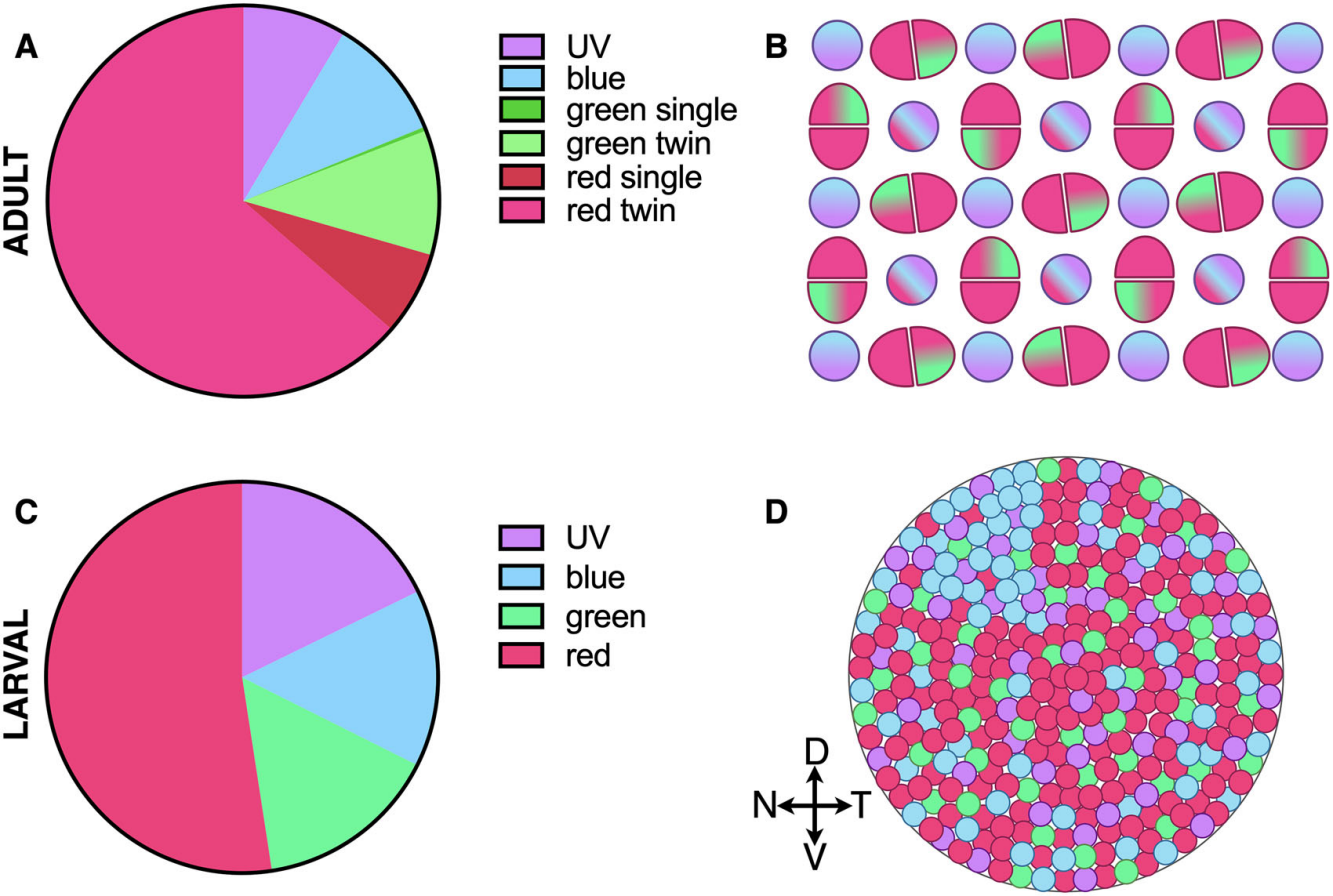
Summary of the cone densities and mosaics for adult and larval turquoise killifish. (A) The proportion of UV, blue, green single, green twin, red single, and red twin cones in adult killifish. The adult killifish cone mosaic is dominated by red cones, especially double red cones. (B) Adult killifish have a square cone mosaic. The double cones forming the mosaic always have a red cone unit, with the other unit being either red or green. The single cones within the center are typically UV, blue, or red cones, while the single cones that can be found at the square corners are blue or UV. (C) The proportion of UV, blue, green, and red cones in the larval killifish retina. (D) The larval cone mosaic has regionalization, with a dorsonasal region of increased blue and UV cone density.

This is the first report of the cone photoreceptor subtypes and mosaic organization in the turquoise killifish, an annual killifish species that lives in turbid waters. Turquoise killifish cone mosaics differ between larvae and adults (Figure 13). Larval fish have a more cone‐enriched photoreceptor population, as is commonly observed in larval fish retinas (Sandy and Blaxter 1980; Pankhurst 1983; Evans et al. 1993; Tohya et al. 1999; Helvik et al. 2001a; Fadool 2003; Allison et al. 2010; Lu et al. 2023), but the proportion of red cones within their cone population is reduced compared to adults. Larval turquoise killifish were found to have regionalization that included a short‐wavelength cone‐rich region in the dorsonasal retina, with an especially high concentration of blue cones. The dorsonasal region of the retina is positioned to visualize the bed of the ephemeral pools that turquoise killifish live in (Valenzano et al. 2015; Kim et al. 2016). It is possible that the increased blue cone density is helpful for visualizing prey from below or shadows of predators from above. Whether the larval dorsonasal blue cone‐dense region provides improved acuity, similar to the zebrafish larvae UV cone‐based high‐acuity zone (Yoshimatsu et al. 2020), is unknown currently.

In contrast to the larvae, adult turquoise killifish have an ordered mosaic in a square formation—however, there is no clear, predictable pattern that the cone subtypes follow within the square mosaic. The single cones found within the squares express UV, blue, red, or, very rarely, green cone opsin, while single cones in square corners express either UV or blue cone opsin. Double cones always contain a red cone unit, but the other unit can be either red or green. This is distinct from zebrafish, which have a row mosaic with highly repeating organization comprised of UV and blue ones alternating within rows and red and green double cones alternating within neighboring rows (Tohya et al. 1999, 2003; Raymond and Barthel 2004; Allison et al. 2010). Why the ordered mosaics of some fishes are comprised of rigid patterns while others have stochasticity is unclear. However, it is known that some fish species have plasticity within their opsin expression. Some species can have different abundances of cone photoreceptor subtypes depending on the light environment in which they were raised, and can even have rapid upregulation of opsin genes upon exposure to different light environments (Fuller et al. 2003, 2005; Fuller and Claricoates 2011; Novales Flamarique et al. 2013; Nandamuri et al. 2017; Luehrmann et al. 2018); it is therefore possible that species without predictable repeating patterns of cone photoreceptors within the ordered mosaic may have more inherent plasticity. Alternatively, the environmental pressures that facilitate a rigid repeating pattern of cone subtypes, which is not understood, may simply be absent from some species, resulting in stochasticity.

This work did not investigate spatial and temporal differences in opsin gene orthologue expression. Fish can express different opsin genes at different life stages and in different retinal regions (Raymond et al. 1993; Robinson et al. 1995; Vihtelic et al. 1999a; Takechi and Kawamura 2005; Tsujimura et al. 2015); it is therefore possible that specific *rh2* or *lws* genes may be enriched at different life stages, within certain retinal regions, and/or between single versus double cones. Future studies can assess the spatial and temporal expression of opsin orthologues in the killifish retina.

The cone types expressing red and green opsin differ between species. Red and green opsins within the adult zebrafish retina are restricted to double cones (Tohya et al. 1999, 2003; Raymond and Barthel 2004; Allison et al. 2010)—however, goldfish, another diurnal fish with high turbidity tolerance, have both red double and red single cones (Marc and Sperling 1976; Ross et al. 1976; Stell and Hárosi 1976), similar to what was observed here in adult killifish. Green single cones are also found in the retina of Atlantic halibut, a deep‐water flatfish (Bolstad and Novales Flamarique 2022, 2023). Why some but not all fish species retain red and green single cones into adulthood is unclear, although it is likely influenced by the light environment. Single red and green cones have only been reported in adult teleosts with non‐row mosaics, which have a larger proportion of medium‐ and long‐wavelength cones overall compared to row mosaics; however, it is worth noting that many previous studies reported simply double versus single cones and did not explore spectral sensitivity or opsin expression to identify subtypes (Eigenmann and Shafer 1900; Lyall 1956, 1957; Engström 1960, 1961, 1963a, 1963b; Collin et al. 1996; Reckel et al. 2002; Collin and Shand 2008), and there may be species with red or green single cones in row mosaics. Square mosaics may be an adaptation for environments with more long‐wavelength light, such as turbid waters where there is filtering of short‐wavelength light, and red or green single cones may be a further way to optimize medium‐ and long‐wavelength vision in animals that are even more heavily reliant on visual information derived from longer‐wavelength opsins.

Few killifish species have been studied for photoreceptor organization, but square cone mosaics are a common feature of those that have been assessed. The striped panchax (*Aplocheilus lineatus*) and bayou topminnow (*Fundulus nottii*), non‐annual killifish that prefer clear waters, were both reported to have square cone mosaics with double cones containing units of equal size (Engström 1963a), as has been observed here with turquoise killifish. Photoreceptors have also been investigated in turbid water‐dwelling non‐annual killifish species. Bluefin killifish (*Lucania goodei*) were reported to have variation in their photoreceptor density depending on whether they were reared in clear or murky waters, and that both genetic and environmental factors influence opsin expression (Fuller et al. 2003, 2005, 2010; Fuller and Claricoates 2011). Fish raised in turbid swamp environments had a higher frequency of red opsin than fish raised in clear springs. However, the bluefin killifish cone mosaic organization has not been characterized, nor whether changes in opsin expression represent co‐expression of multiple opsins within a single cell or cell identity switches. Cone subtypes and mosaic organization have been assessed for the mummichog killifish (*Fundulus heteroclitus*), a species that also lives in turbid waters (Butcher 1938; Novales Flamarique and Hárosi 2000; Suliman and Novales Flamarique 2014). The mummichog cone mosaic contains both squares and rows (Novales Flamarique and Hárosi 2000; Suliman and Novales Flamarique 2014). In the mummichog row mosaic, UV and blue cones alternate between double cone rows (Suliman and Novales Flamarique 2014). However, the overall photoreceptor subtype proportions and regularity were not assessed. Nonetheless, square mosaics have a higher proportion of red/green double cones than row mosaics, and the mummichog retina is likely to have an abundance of red cones as a result. It is unknown whether it is common for killifish species to lack regularity in spatial orientation of the cone subtypes within their mosaics. Furthermore, cone mosaics have not been investigated in the striped panchax, bayou topminnow, bluefin killifish, nor mummichog killifish larvae—it is thus not known whether a short‐wavelength cone‐dense region is a specialization of turquoise killifish or a feature of larval killifishes.

Genetics contributes to the proportion of cones within the mosaic, leading to phenotypic variability between individuals. This has been observed not only in fishes (Fuller et al. 2005; Novales Flamarique et al. 2013), but also in humans (Gunther et al. 2008). While the phenomenon of heritability has been observed in threespine sticklebacks and bluefin killifish (Fuller et al. 2005; Novales Flamarique et al. 2013), the genetic sources have not been identified. In humans, however, single‐nucleotide polymorphisms upstream from the red/green (long/medium; L/M) cone opsin array have been associated with different cone subtype abundance phenotypes (Gunther et al. 2008). Here, variation between individual turquoise killifish was observed, but whether there are discrete phenotypes controlled by genetic mechanisms is not known. While lab‐grown killifish lines are inbred, genetic or epigenetic variation between individuals can nonetheless lead to variable phenotypes. Conversely, environmental or stochastic mechanisms may be contributing to cone subtype abundance and organization in the turquoise killifish cone mosaic.

## 5 Conclusion

Annual killifishes have unique evolutionary adaptations to allow them to thrive in seasonal waters of varying composition and turbidity. The cone mosaic in the turquoise killifish contains many features observed in other fishes adapted for living in turbid waters, including red single cones, an overall abundance of red cones across life stages, and a square cone mosaic in adulthood. The ordered square mosaic lacks cone subtype co‐regularity across the retina, demonstrating cone subtype randomness within the square mosaic. Larvae have a dorsonasal area of unknown function with increased density of short‐wavelength cones. This study was the first to characterize the cone mosaic in an annual turquoise killifish species; exploration of additional species is needed to determine whether these features are common amongst annual killifishes or unique to the turquoise killifish.

## Data Availability

The data that support the findings of this study are available from the corresponding author upon reasonable request.

## References

[cne70199-bib-0001] Allison, W. T. , L. K. Barthel , K. M. Skebo , M. Takechi , S. Kawamura , and P. A. Raymond . 2010. “Ontogeny of Cone Photoreceptor Mosaics in Zebrafish.” Journal of Comparative Neurology 518: 4182–4195. http://www.ncbi.nlm.nih.gov/pubmed/20878782. 20878782 10.1002/cne.22447 PMC3376642

[cne70199-bib-0002] Archer, S. , A. Hope , and J. C. Partridge . 1995. “The Molecular Basis for the Green‐Blue Sensitivity Shift in the Rod Visual Pigments of the European Eel.” Proceedings of the Royal Society B 262: 289–295. https://royalsocietypublishing.org/. 8587887 10.1098/rspb.1995.0208

[cne70199-bib-0003] Beaudet, L. , I. N. Flamarique , and C. W. Hawryshyn . 1997. “Cone Photoreceptor Topography in the Retina of Sexually Mature Pacific Salmonid Fishes.” Journal of Comparative Neurology 383: 49–59. 9184985

[cne70199-bib-0004] Bergmans, S. , N. C. L. Noel , L. Masin , et al. 2024. “Age‐Related Dysregulation of the Retinal Transcriptome in African Turquoise Killifish.” Aging Cell 23: e14192. 38742929 10.1111/acel.14192 PMC11320354

[cne70199-bib-0006] Bolstad, K. , and I. Novales Flamarique . 2022. “Photoreceptor Distributions, Visual Pigments and the Opsin Repertoire of Atlantic Halibut (*Hippoglossus hippoglossus*).” Scientific Reports 12: 8062. 35577858 10.1038/s41598-022-11998-9 PMC9110347

[cne70199-bib-0007] Bolstad, K. , and I. Novales Flamarique . 2023. “Chromatic Organization of Retinal Photoreceptors During Eye Migration of Atlantic Halibut (*Hippoglossus hippoglossus*).” Journal of Comparative Neurology 531: 256–280. 36217253 10.1002/cne.25423

[cne70199-bib-0008] Butcher, E. O. 1938. “The Structure of the Retina of *Fundulus heteroclitus* and the Regions of the Retina Associated With the Different Chromatophoric Responses.” Journal of Experimental Zoology 79: 275–297.

[cne70199-bib-0009] Carleton, K. L. , D. Escobar‐Camacho , S. M. Stieb , F. Cortesi , and N. J. Marshall . 2020. “Seeing the Rainbow: Mechanisms Underlying Spectral Sensitivity in Teleost Fishes.” Journal of Experimental Biology 223: jeb193334. 32327561 10.1242/jeb.193334 PMC7188444

[cne70199-bib-0010] Chen, J. , R. C. Khondker , and A. Brunet . 2023. “Breeding and Reproduction of the African Turquoise Killifish *Nothobranchius furzeri* .” Cold Spring Harbor Protocols 2023: pdb.prot107816. 10.1101/pdb.prot107816 36863853

[cne70199-bib-0011] Cheng, C. L. , and I. N. Flamarique . 2007. “Chromatic Organization of Cone Photoreceptors in the Retina of Rainbow Trout: Single Cones Irreversibly Switch From UV (SWS1) to Blue (SWS2) Light Sensitive Opsin During Natural Development.” Journal of Experimental Biology 210: 4123–4135. 18025012 10.1242/jeb.009217

[cne70199-bib-0012] Choi, H. M. T. , M. Schwarzkopf , M. E. Fornace , et al. 2018. “Third‐Generation In Situ Hybridization Chain Reaction: Multiplexed, Quantitative, Sensitive, Versatile, Robust.” Development 145: dev165753. 29945988 10.1242/dev.165753 PMC6031405

[cne70199-bib-0013] Collin, S. P. , H. B. Collin , and M. A. Ali . 1996. “Fine Structure of the Retina and Pigment Epithelium in the Creek Chub, *Semotilus atromaculatus* (Cyprinidae, Teleostei).” Histology and Histopathology 11: 41–53. http://www.hh.um.es. 8720447

[cne70199-bib-0014] Collin, S. P. , and J. Shand . 2008. “Retinal Sampling and the Visual Field in Fishes.” In Sensory Processing in Aquatic Environments, 139–169. Springer.

[cne70199-bib-0015] De Busserolles, F. , F. Cortesi , L. Fogg , S. M. Stieb , M. Luehrmann , and N. J. Marshall . 2021. “The Visual Ecology of Holocentridae, a Nocturnal Coral Reef Fish Family With a Deep‐Sea‐Like Multibank Retina.” Journal of Experimental Biology 224: jeb233098. 33234682 10.1242/jeb.233098

[cne70199-bib-0016] Downing, J. E. G. , M. B. A. Djamgoz , and J. K. Bowmaker . 1986. “Photoreceptors of a Cyprinid Fish, the Roach: Morphological and Spectral Characteristics.” Journal of Comparative Physiology A 159: 859–868.

[cne70199-bib-0017] Easter, S. S. , and D. A. Cameron . 1993. “The Cone Photoreceptor Mosaic of the Green Sunfish, *Lepomis cyanellus* .” Visual Neuroscience 10: 375–384. 8485099 10.1017/s095252380000376x

[cne70199-bib-0018] Eigenmann, C. H. , and G. D. Shafer . 1900. “The Mosaic of Single and Twin Cones in the Retina of Fishes.” American Naturalist 34: 398.

[cne70199-bib-0019] Engström, K. 1960. “Cone Types and Cone Arrangement in the Retina of Some Cyprinids.” Acta Zoologica 41: 277–295.

[cne70199-bib-0020] Engström, K. 1961. “Cone Types and Cone Arrangement in the Retina of Some Gadids.” Acta Zoologica 42: 227–243.

[cne70199-bib-0021] Engström, K. 1963a. “Cone Types and Cone Arrangements in Teleost Retinae.” Acta Zoologica 44: 179–243.

[cne70199-bib-0022] Engström, K. 1963b. “Structure, Organization and Ultrastructure of the Visual Cells in the Teleost Family Labrida.” Acta Zoologica 44: 1–41.

[cne70199-bib-0023] Engström, K. , and I.‐B. Ahlbert . 1963. “Cone Types and Cone Arrangement in the Retina of Some Flatfishes.” Acta Zoologica 44: 119–129.

[cne70199-bib-0024] Evans, B. I. , F. I. Hárosi , and R. D. Fernald . 1993. “Photoreceptor Spectral Absorbance in Larval and Adult Winter Flounder (*Pseudopleuronectes americanus*).” Visual Neuroscience 10: 1065–1071. 8257663 10.1017/s0952523800010178

[cne70199-bib-0025] Fadool, J. M. 2003. “Development of a Rod Photoreceptor Mosaic Revealed in Transgenic Zebrafish.” Developmental Biology 258: 277–290. 12798288 10.1016/s0012-1606(03)00125-8

[cne70199-bib-0026] Fleisch, V. C. , T. Jametti , and S. C. F. Neuhauss . 2008. “Electroretinogram (ERG) Measurements in Larval Zebrafish.” Cold Spring Harbor Protocols 3: 1–6. 10.1101/pdb.prot4973 21356789

[cne70199-bib-0027] Frau, S. , I. Novales Flamarique , P. W. Keeley , B. E. Reese , and J. A. Muñoz‐Cueto . 2020. “Straying From the Flatfish Retinal Plan: Cone Photoreceptor Patterning in the Common Sole (*Solea solea*) and the Senegalese Sole (*Solea senegalensis*).” Journal of Comparative Neurology 528: 2283–2307. 32103501 10.1002/cne.24893 PMC7392819

[cne70199-bib-0028] Fuller, R. C. , K. L. Carleton , J. M. Fadool , T. C. Spady , and J. Travis . 2005. “Genetic and Environmental Variation in the Visual Properties of Bluefin Killifish, *Lucania goodei* .” Journal of Evolutionary Biology 18: 516–523. 15842481 10.1111/j.1420-9101.2005.00886.x

[cne70199-bib-0029] Fuller, R. C. , and K. M. Claricoates . 2011. “Rapid Light‐Induced Shifts in Opsin Expression: Finding New Opsins, Discerning Mechanisms of Change, and Implications for Visual Sensitivity.” Molecular Ecology 20: 3321–3335. 21749514 10.1111/j.1365-294X.2011.05180.x

[cne70199-bib-0030] Fuller, R. C. , L. J. Fleishman , M. Leal , J. Travis , and E. Loew . 2003. “Intraspecific Variation in Retinal Cone Distribution in the Bluefin Killifish, *Lucania goodei* .” Journal of Comparative Physiology A: Sensory, Neural, and Behavioral Physiology 189: 609–616. 10.1007/s00359-003-0435-x 12879350

[cne70199-bib-0031] Fuller, R. C. , L. A. Noa , and R. S. Strellner . 2010. “Teasing Apart the Many Effects of Lighting Environment on Opsin Expression and Foraging Preference in Bluefin Killifish.” American Naturalist 176: 1–13. 10.1086/652994 20497054

[cne70199-bib-0032] Gunther, K. L. , J. Neitz , and M. Neitz . 2008. “Nucleotide Polymorphisms Upstream of the X‐Chromosome Opsin Gene Array Tune L:M Cone Ratio.” Visual Neuroscience 25: 265–271. 18598397 10.1017/S0952523808080280 PMC2999001

[cne70199-bib-0033] Helvik, J. V. , Ø. Drivenes , T. Harboe , and H.‐C. Seo . 2001a. “Topography of Different Photoreceptor Cell Types in the Larval Retina of Atlantic Halibut (*Hippoglossus hippoglossus*).” Journal of Experimental Biology 204: 2553–2559. 11511671 10.1242/jeb.204.14.2553

[cne70199-bib-0034] Helvik, J. V. , Ø. Drivenes , T. H. Næss , A. Fjose , and H. C. Seo . 2001b. “Molecular Cloning and Characterization of Five Opsin Genes From the Marine Flatfish Atlantic Halibut (*Hippoglossus hippoglossus*).” Visual Neuroscience 18: 767–780. 11925012 10.1017/s095252380118510x

[cne70199-bib-0035] Hofer, H. , J. Carroll , J. Neitz , M. Neitz , and D. R. Williams . 2005. “Organization of the human Trichromatic Cone Mosaic.” Journal of Neuroscience 25: 9669–9679. 16237171 10.1523/JNEUROSCI.2414-05.2005 PMC6725723

[cne70199-bib-0036] Hu, H. , Y. Qin , Z. Qu , et al. 2024. “The Zpr‐3 Antibody Recognizes the 320–354 Region of Rho and Labels both Rods and Green Cones in Zebrafish.” Zebrafish 21: 394–400. 39316468 10.1089/zeb.2024.0159

[cne70199-bib-0037] Kim, Y. , H. G. Nam , and D. R. Valenzano . 2016. “The Short‐Lived African Turquoise Killifish: An Emerging Experimental Model for Ageing.” Disease Models & Mechanisms 9: 115–129. 26839399 10.1242/dmm.023226 PMC4770150

[cne70199-bib-0038] Larison, K. D. , and R. Bremiller . 1990. “Early Onset of Phenotype and Cell Patterning in the Embryonic Zebrafish Retina.” Development 109: 567–576. 2401210 10.1242/dev.109.3.567

[cne70199-bib-0039] Lu, K. , X. F. Liang , S. L. Tang , et al. 2023. “Role of Short‐Wave‐Sensitive 1 (sws1) in Cone Development and First Feeding in Larval Zebrafish.” Fish Physiology and Biochemistry 49: 801–813. 37495865 10.1007/s10695-023-01213-5

[cne70199-bib-0040] Luehrmann, M. , S. M. Stieb , K. L. Carleton , A. Pietzker , K. L. Cheney , and N. J. Marshall . 2018. “Short‐Term Colour Vision Plasticity on the Reef: Changes in Opsin Expression Under Varying Light Conditions Differ Between Ecologically Distinct Fish Species.” Journal of Experimental Biology 221: jeb175281. 30158132 10.1242/jeb.175281

[cne70199-bib-0041] Lyall, A. H. 1956. “Occurrence of Triple and Quadruple Cones in the Retina of the Minnow (*Phoxinus laevis*).” Nature 177: 1086–1087.

[cne70199-bib-0042] Lyall, A. H. 1957. “Cone Arrangements in Teleost Retinae.” Quarterly Journal of Microscopical Science 98: 189–201.

[cne70199-bib-0043] Marc, R. E. , and H. G. Sperling . 1976. “The Chromatic Organization of the Goldfish Cone Mosaic.” Vision Research 16: 1211–1224. 1006992 10.1016/0042-6989(76)90044-4

[cne70199-bib-0044] McEwan, M. R. 1938. “A Comparison of the Retina of the Mormyrids With That of Various Other Teleosts.” Acta Zoologica 19: 427–465.

[cne70199-bib-0045] Mokhtar, D. M. , M. Albano , R. Alonaizan , and A. Attaai . 2024. “Retinal Structure of *Poecilia sphenops*: Photoreceptor Mosaics, Synaptic Ribbon Patterns, and Glial Cell Expressions.” Animals 14: 939. 38540038 10.3390/ani14060939 PMC10967514

[cne70199-bib-0046] Moretti, B. , S. N. Rodriguez Alvarez , and H. E. Grecco . 2023. “Nfinder: Automatic Inference of Cell Neighborhood in 2D and 3D Using Nuclear Markers.” BMC Bioinformatics 24: 230. 37270479 10.1186/s12859-023-05284-2 PMC10239575

[cne70199-bib-0047] Nandamuri, S. P. , M. R. Yourick , and K. L. Carleton . 2017. “Adult Plasticity in African Cichlids: Rapid Changes in Opsin Expression in Response to Environmental Light Differences.” Molecular Ecology 26: 6036–6052. 28926160 10.1111/mec.14357 PMC5690868

[cne70199-bib-0048] Nawrocki, L. , R. Bremiller , G. Streisinger , and M. Kaplan . 1985. “Larval and Adult Visual Pigments of the Zebrafish, *Brachydanio rerio* .” Vision Research 25: 1569–1576. 3832580 10.1016/0042-6989(85)90127-0

[cne70199-bib-0049] Nguyen, L. T. , H. A. Schmidt , A. Von Haeseler , and B. Q. Minh . 2015. “IQ‐TREE: A Fast and Effective Stochastic Algorithm for Estimating Maximum‐Likelihood Phylogenies.” Molecular Biology and Evolution 32: 268–274. 25371430 10.1093/molbev/msu300 PMC4271533

[cne70199-bib-0050] Nishiwaki, Y. , T. Oishi , F. Tokunaga , and T. Morita . 1997. “Three‐Dimensional Reconstitution of Cone Arrangement on the Spherical Surface of the Retina in the Medaka Eyes.” Zoological Science 14: 795–801.

[cne70199-bib-0051] Noel, N. C. L. , and W. T. Allison . 2018. “Connectivity of Cone Photoreceptor Telodendria in the Zebrafish Retina.” Journal of Comparative Neurology 526: 609–625. 29127712 10.1002/cne.24354

[cne70199-bib-0052] Novales Flamarique, I. , C. L. Cheng , C. Bergstrom , and T. E. Reimchen . 2013. “Pronounced Heritable Variation and Limited Phenotypic Plasticity in Visual Pigments and Opsin Expression of Threespine Stickleback Photoreceptors.” Journal of Experimental Biology 216: 656–667. 23077162 10.1242/jeb.078840

[cne70199-bib-0053] Novales Flamarique, I. , and F. I. Hárosi . 2000. “Photoreceptors, Visual Pigments, and Ellipsosomes in the Killifish, *Fundulus heteroclitus*: A Microspectrophotometric and Histological Study.” Visual Neuroscience 17: 403–420. 10910108 10.1017/s0952523800173080

[cne70199-bib-0055] Pankhurst, N. W. 1983. “Retinal Development in Larval and Juvenile European Eel, *Anguilla anguilla* (L.).” Canadian Journal of Zoology 62: 335–343. http://www.nrcresearchpress.com.

[cne70199-bib-0057] Pointer, M. A. , C. H. C. Cheng , J. K. Bowmaker , et al. 2005. “Adaptations to an Extreme Environment: Retinal Organisation and Spectral Properties of Photoreceptors in Antarctic Notothenioid Fish.” Journal of Experimental Biology 208: 2363–2376. 15939776 10.1242/jeb.01647

[cne70199-bib-0058] Raymond, P. A. , and L. K. Barthel . 2004. “A Moving Wave Patterns the Cone Photoreceptor Mosaic Array in the Zebrafish Retina.” International Journal of Developmental Biology 48: 935–945. http://www.ncbi.nlm.nih.gov/pubmed/15558484. 15558484 10.1387/ijdb.041873pr

[cne70199-bib-0059] Raymond, P. A. , L. K. Barthel , M. E. Rounsifer , S. A. Sullivan , and J. K. Knight . 1993. “Expression of Rod and Cone Visual Pigments in Goldfish and Zebrafish: A Rhodopsin‐Like Gene Is Expressed in Cones.” Neuron 10: 1161–1174. 8318234 10.1016/0896-6273(93)90064-x

[cne70199-bib-0060] Reckel, F. , R. R. Melzer , J. W. L. Parry , and J. K. Bowmaker . 2002. “The Retina of Five Atherinomorph Teleosts: Photoreceptors, Patterns and Spectral Sensitivities.” Brain, Behavior and Evolution 60: 249–264. 12476052 10.1159/000067191

[cne70199-bib-0061] Robinson, J. , E. A. Schmitt , and J. E. Dowling . 1995. “Temporal and Spatial Patterns of Opsin Gene Expression in Zebrafish (*Danio rerio*).” Visual Neuroscience 12: 895–906. 8924413 10.1017/s0952523800009457

[cne70199-bib-0062] Ross, W. N. , D. Landowne , A. B. Waggoner , et al. 1976. “Color Receptor Identities of Goldfish Cones.” Science 191: 487–489. https://www.science.org. 1246634 10.1126/science.1246634

[cne70199-bib-0063] Rossetto, E. S. , H. Dolder , and I. Sazima . 1992. “Double Cone Mosaic Pattern in the Retina of Larval and Adult Piranha, *Serrasalmus spilopleura* .” Experientia 48: 597–599.

[cne70199-bib-0064] Samy, C. , and J. Hirsch . 1989. “Comparison of human and Monkey Retinal Photoreceptor Sampling Mosaics.” Visual Neuroscience 3: 281–285. 2487108 10.1017/s0952523800010038

[cne70199-bib-0065] Sandy, J. M. , and J. H. S. Blaxter . 1980. “A Study of Retinal Development in Larval Herring and Sole.” Journal of the Marine Biological Association of the United Kingdom 60: 59–71.

[cne70199-bib-0066] Stell, W. K. , and F. I. Hárosi . 1976. “Cone Structure and Visual Pigment Content in the Retina of the Goldfish.” Vision Research 16: 647–657. 960588 10.1016/0042-6989(76)90013-4

[cne70199-bib-0067] Suliman, T. , and I. Novales Flamarique . 2014. “Visual Pigments and Opsin Expression in the Juveniles of Three Species of Fish (Rainbow Trout, Zebrafish, and Killifish) Following Prolonged Exposure to Thyroid Hormone or Retinoic Acid.” Journal of Comparative Neurology 522: 98–117. 23818308 10.1002/cne.23391

[cne70199-bib-0068] Takechi, M. , and S. Kawamura . 2005. “Temporal and Spatial Changes in the Expression Pattern of Multiple Red and Green Subtype Opsin Genes During Zebrafish Development.” Journal of Experimental Biology 208: 1337–1345. 15781894 10.1242/jeb.01532

[cne70199-bib-0069] Tohya, S. , A. Mochizuki , and Y. Iwasa . 1999. “Formation of Cone Mosaic of Zebrafish Retina.” Journal of Theoretical Biology 200: 231–244. 10.1006/jtbi.1999.0990. 10504288

[cne70199-bib-0070] Tohya, S. , A. Mochizuki , and Y. Iwasa . 2003. “Difference in the Retinal Cone Mosaic Pattern Between Zebrafish and Medaka: Cell‐Rearrangement Model.” Journal of Theoretical Biology 221: 289–300. 12628235 10.1006/jtbi.2003.3192

[cne70199-bib-0071] Tsujimura, T. , R. Masuda , R. Ashino , and S. Kawamura . 2015. “Spatially Differentiated Expression of Quadruplicated Green‐Sensitive RH2 Opsin Genes in Zebrafish Is Determined by Proximal Regulatory Regions and Gene Order to the Locus Control Region.” BMC Genetics 16: 130. 26537431 10.1186/s12863-015-0288-7 PMC4634787

[cne70199-bib-0072] Valenzano, D. R. , B. A. Benayoun , P. P. Singh , et al. 2015. “The African Turquoise Killifish Genome Provides Insights Into Evolution and Genetic Architecture of Lifespan.” Cell 163: 1539–1554. 26638078 10.1016/j.cell.2015.11.008 PMC4684691

[cne70199-bib-0073] Viets, K. , K. C. Eldred , and R. J. Johnston . 2016. “Mechanisms of Photoreceptor Patterning in Vertebrates and Invertebrates.” Trends in Genetics 32: 638–659. 10.1016/j.tig.2016.07.004. 27615122 PMC5035628

[cne70199-bib-0074] Vihtelic, T. S. , C. J. Doro , and D. R. Hyde . 1999a. “Cloning and Characterization of Six Zebrafish Photoreceptor Opsin cDNAs and Immunolocalization of Their Corresponding Proteins.” Visual Neuroscience 16: 571–585. 10349976 10.1017/s0952523899163168

[cne70199-bib-0075] Vihtelic, T. S. , C. J. Doro , and D. R. Hyde . 1999b. “Cloning and Characterization of Six Zebrafish Photoreceptor Opsin cDNAs and Immunolocalization of Their Corresponding Proteins.” Visual Neuroscience 16: 571–585. 10349976 10.1017/s0952523899163168

[cne70199-bib-0076] Yin, J. , J. Brocher , B. Linder , et al. 2012. “The 1D4 Antibody Labels Outer Segments of Long Double Cone but Not Rod Photoreceptors in Zebrafish.” Investigative Ophthalmology & Visual Science 53: 4943. 22743318 10.1167/iovs.12-9511

[cne70199-bib-0077] Yoshimatsu, T. , C. Schröder , N. E. Nevala , P. Berens , and T. Baden . 2020. “Fovea‐Like Photoreceptor Specializations Underlie Single UV Cone Driven Prey‐Capture Behavior in Zebrafish.” Neuron 107: 320–337.e6. 32473094 10.1016/j.neuron.2020.04.021 PMC7383236

